# Supplementary figures and images for: A predictive framework for identifying source populations of non-native marine macroalgae: Chondria tumulosa in the Pacific Ocean
Source: PeerJ. 2025 Jun 23;13:e19610. doi: 10.7717/peerj.19610 (PMC12199741; doi:10.7717/peerj.19610)

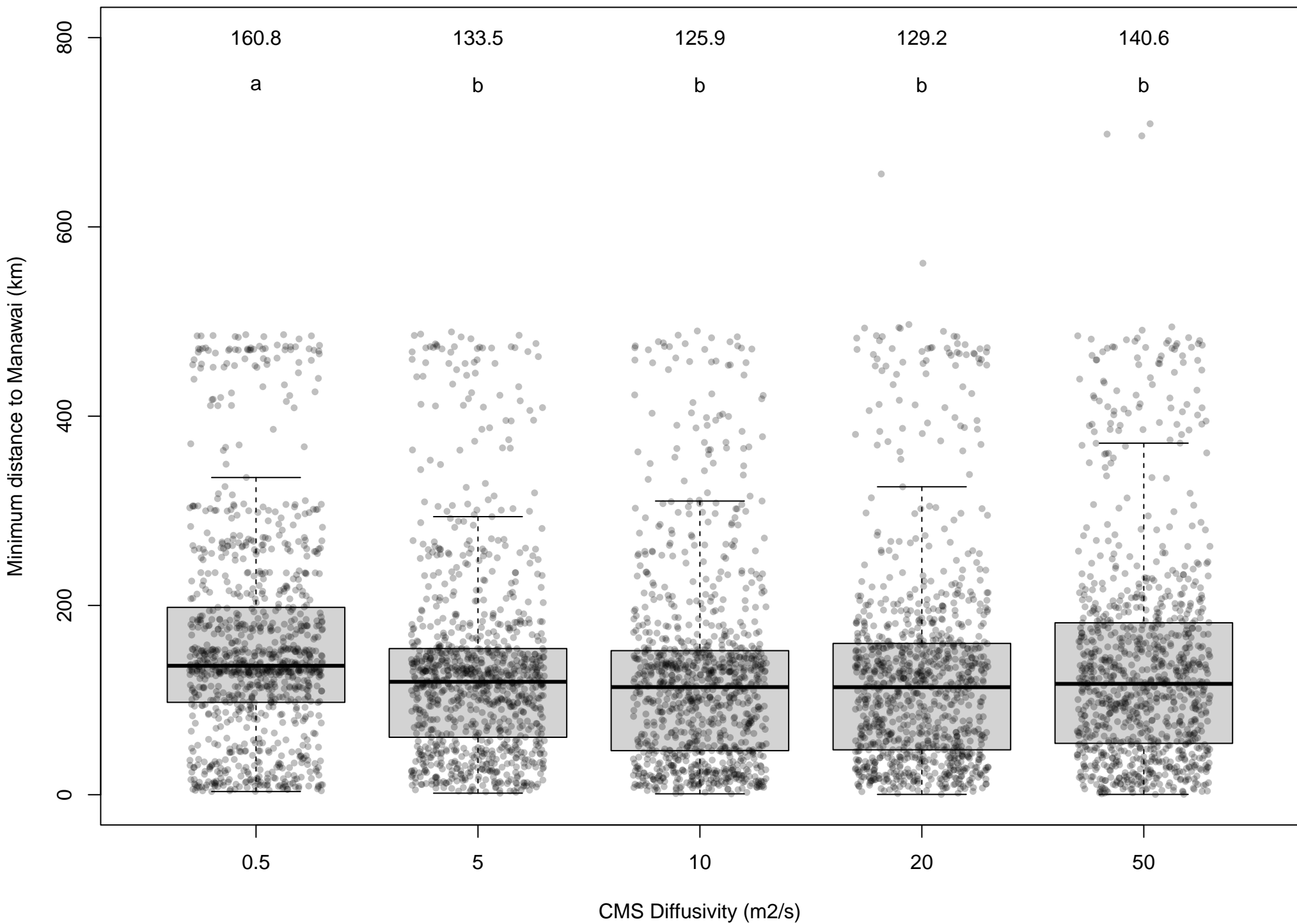

Supplement: Supplemental Information 1 — The final GPS coordinates for satellite-tagged marine debris objects originating at Manawai, Hawai‘i were designated as the origin for a series of targeted CMS model runs. For each run, 1000 particles were backtracked and their minimum distance to Manawai recorded and displayed using a jitter function alongside the boxplot representing the model run. Each model run varied only in the horizontal diffusivity value provided to CMS at 0.5, 5, 10, 20, and 50 m 2 /s. Mean minimum passing distance (km) to Manawai and significance categories are displayed above their respective diffusivity values. Interquartile ranges (IQRs) for each model run were 97.7-198.0 km (0.5 m 2 /s), 60.7-154.4 km (5 m 2 /s), 46.6-152.2 km (10 m 2 /s), 47.4-159.8 km (20 m 2 /s), and 54.4-181.6 km (50 m 2 /s), with whiskers extending to 1.5*IQR. Dots beyond the whiskers represent outliers. The diffusivity value 10 m 2 /s exhibited the lowest mean passing distance and was selected as the horizontal diffusivity value for the CMS run. Horizontal diffusivities from 5-50 m 2 /s did not vary significantly. [file peerj-13-19610-s001.pdf]

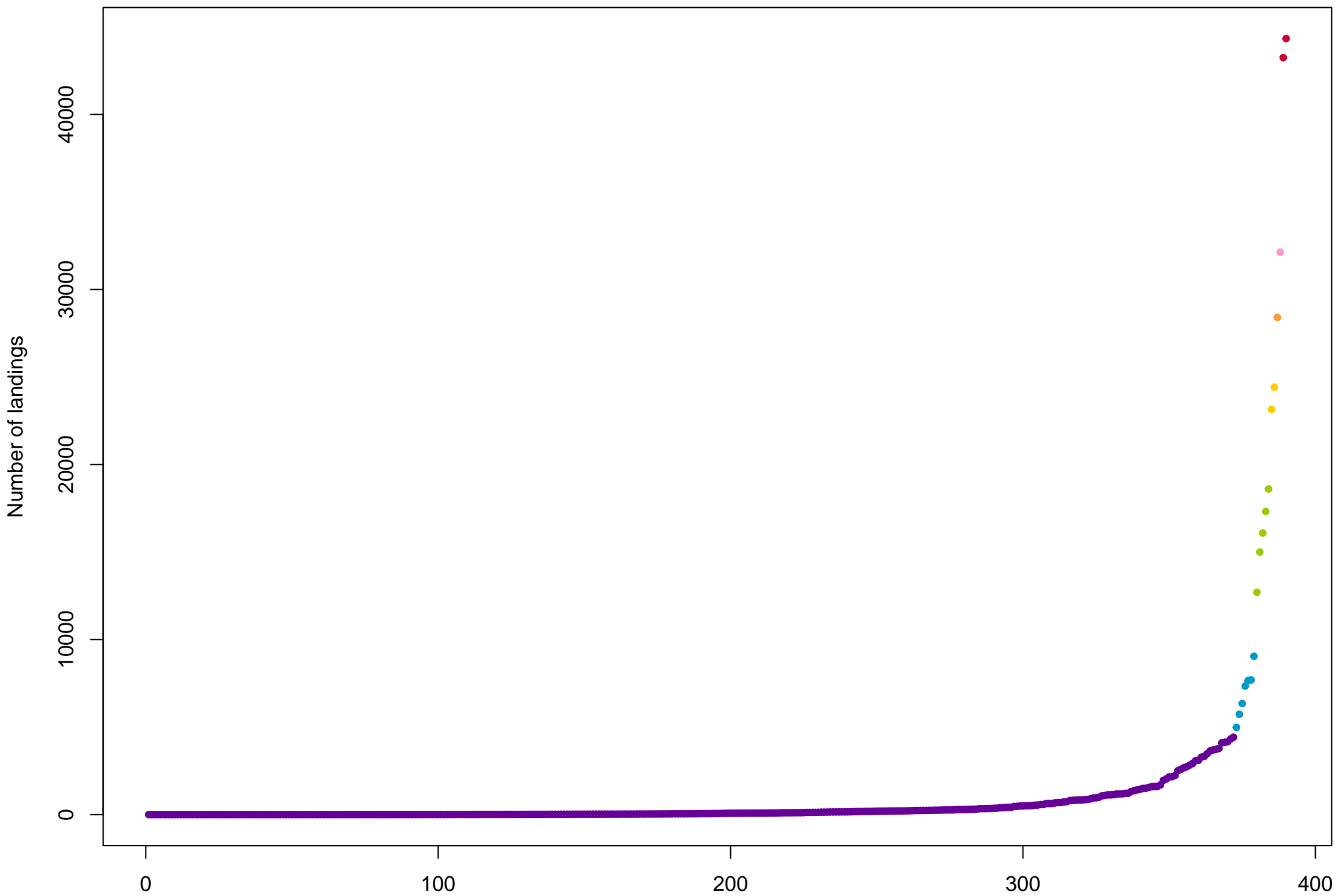

Supplement: Supplemental Information 2 — Landings are colored in accordance with the color key shown in Figure 1. Particles backtracked from Manawai, Hawai‘i were released and tracked from January 1, 2000 to December 30, 2015 until contacting a settlement polygon in the Pacific Ocean. The cutoff between polygons colored purple and light blue is 1% of all settled particles. Most hexagons with landings did not receive many particles while the 18 hexagons of varying colors received many more. [file peerj-13-19610-s002.pdf]

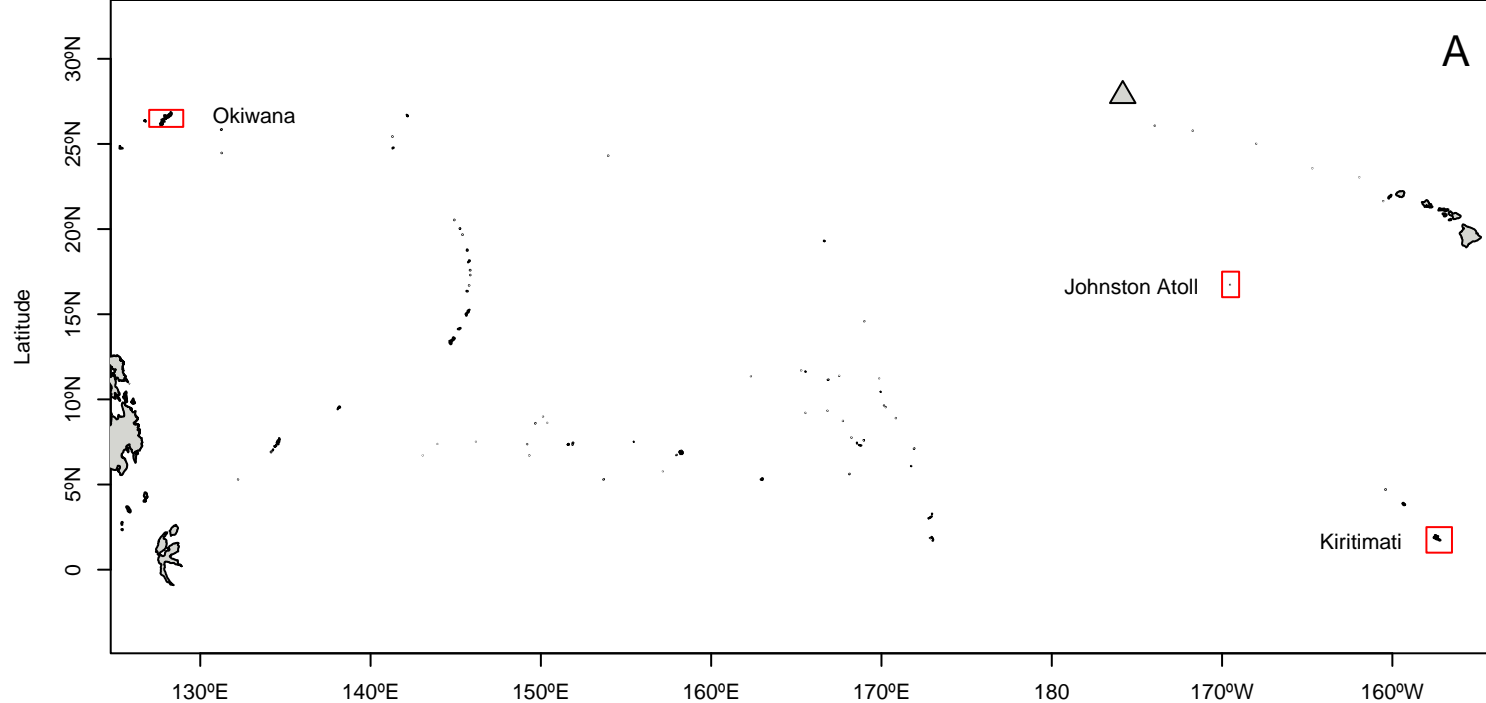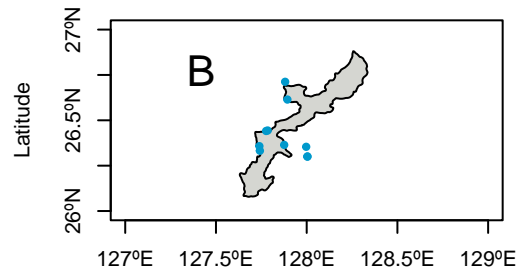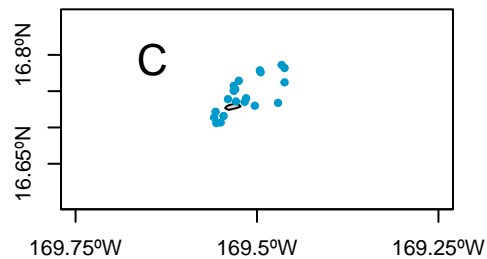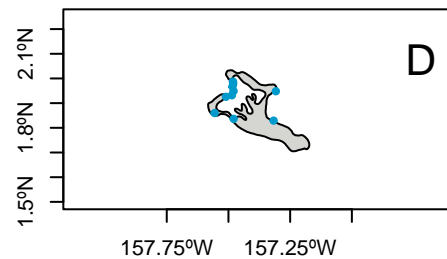

Longitude

Supplement: Supplemental Information 3 — In each region, 2-L duplicate seawater samples were collected from within 1 m of the bottom. To increase screening efficiency, eDNA from replicate samples was pooled within each region and amplified via qPCR. A second pool containing spiked positive eDNA from a known site (Kuaihelani, PMNM) was created to verify assay sensitivity. No sampling locations included a positive hit for C. tumulosa. The red boxes in panel A represent the sampling locations across the Pacific and blue dots in panels B-D represent the individual samples taken. Gray triangle represents the location of Manawai. [file peerj-13-19610-s003.pdf]

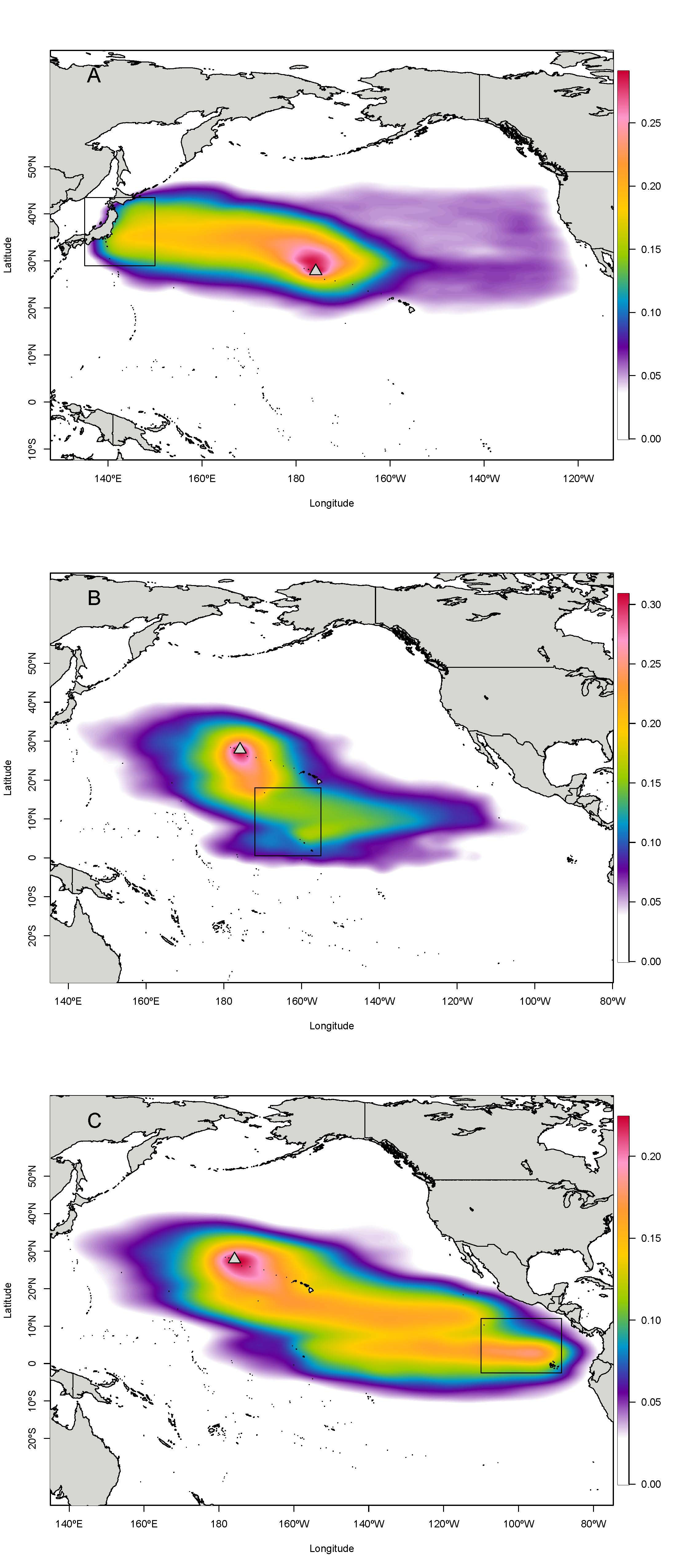

Supplement: Supplemental Information 4 — Legend and colors indicate the proportion of particles located in each position throughout the study period. Particle pathways are shown originating from Japan (A), the central Pacific (B), and the Eastern Tropical Pacific (C) as potential introduction pathways for Chondria tumulosa to Manawai, Hawai‘i. The gray triangle shows the location of Manawai and the box in each panel represents the potential source region. [file peerj-13-19610-s004.jpg]

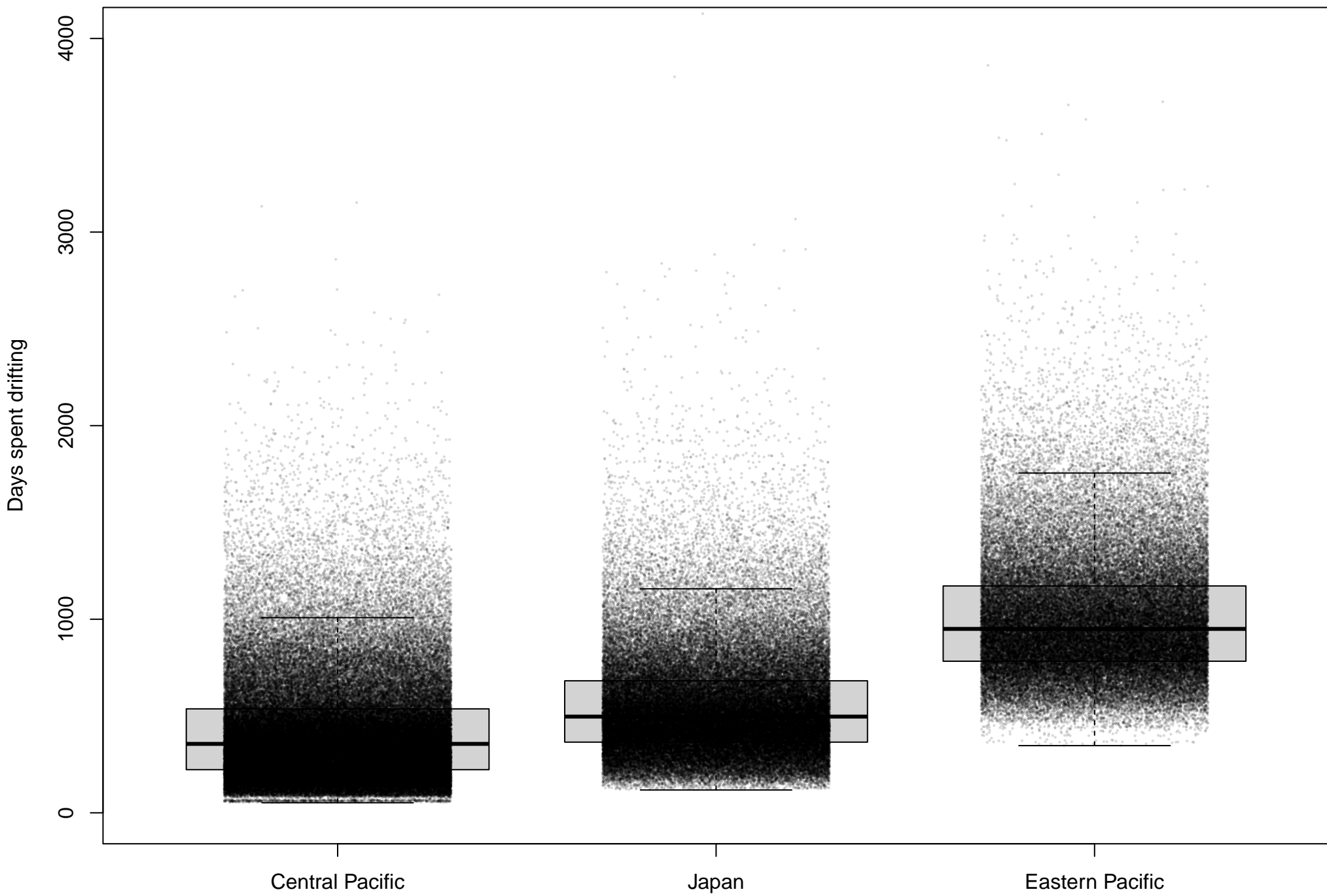

Supplement: Supplemental Information 5 — The drift times for particles settling in the central Pacific, Japan, and the eastern Pacific all exhibit significant differences with mean drifting time increasing from left to right. The median (mean) drift times for the central Pacific, Japan, and eastern Pacific were 356 (420), 497 (552), and 950 (1007) days, respectively. Interquartile ranges (IQRs) for each location were 97.7-198.0 days (central Pacific), 60.7-154.4 days (Japan), and 46.6-152.2 days (eastern Pacific), with whiskers extending to 1.5*IQR. Dots beyond the whiskers represent outliers. [file peerj-13-19610-s005.pdf]
