# Supplementary material for: A predictive framework for identifying source populations of non-native marine macroalgae: Chondria tumulosa in the Pacific Ocean
Source: PeerJ. 2025 Jun 23;13:e19610. doi: 10.7717/peerj.19610 (PMC12199741; doi:10.7717/peerj.19610)
Supplement: Supplemental Information 6 [file peerj-13-19610-s006.rtf]

Site	Region	Samples	Latitude 	Longitude 	Date	
KIR01	Line Islands	2	1.984644	-157.481	3/21/2024	
KIR02	Line Islands	2	1.966633	-157.484	3/21/2024	
KIR03	Line Islands	2	1.829158	-157.317	3/22/2024	
KIR04	Line Islands	2	1.948143	-157.308	3/22/2024	
KIR05	Line Islands	2	1.948787	-157.479	3/23/2024	
KIR06	Line Islands	2	1.976351	-157.481	3/23/2024	
KIR07	Line Islands	2	1.836328	-157.479	3/24/2024	
KIR08	Line Islands	2	1.859773	-157.556	3/24/2024	
KIR09	Line Islands	2	1.93185	-157.486	3/25/2024	
KIR10	Line Islands	2	1.987664	-157.482	3/26/2024	
KIR11	Line Islands	2	1.925672	-157.51	3/26/2024	
J01	Johnston Atoll	2	16.76428	-169.525	7/24/2024	
J02	Johnston Atoll	2	16.75743	-169.532	7/24/2024	
J03	Johnston Atoll	2	16.77871	-169.496	7/24/2024	
J04	Johnston Atoll	2	16.77605	-169.495	7/24/2024	
J05	Johnston Atoll	2	16.78611	-169.466	7/25/2024	
J06	Johnston Atoll	2	16.78189	-169.462	7/25/2024	
J07	Johnston Atoll	2	16.73583	-169.529	7/25/2024	
J08	Johnston Atoll	2	16.73898	-169.54	7/25/2024	
J09	Johnston Atoll	2	16.74023	-169.515	7/26/2024	
J10	Johnston Atoll	2	16.75039	-169.532	7/26/2024	
J11	Johnston Atoll	2	16.72161	-169.557	7/26/2024	
J12	Johnston Atoll	2	16.75275	-169.53	7/26/2024	
J13	Johnston Atoll	2	16.70686	-169.55	7/26/2024	
J14	Johnston Atoll	2	16.71379	-169.559	7/26/2024	
J15	Johnston Atoll	2	16.70594	-169.556	7/26/2024	
J16	Johnston Atoll	2	16.76217	-169.462	7/27/2024	
J17	Johnston Atoll	2	16.73387	-169.471	7/27/2024	
J18	Johnston Atoll	2	16.72991	-169.503	7/27/2024	
J19	Johnston Atoll	2	16.71582	-169.546	7/27/2024	
J20	Johnston Atoll	2	16.73533	-169.517	7/26/2024	
OK01	Japan	2	26.35752	127.7393	6/19/2024	
OK02	Japan	2	26.33215	127.7421	6/19/2024	
OK03	Japan	2	26.44116	127.7764	6/20/2024	
OK04	Japan	2	26.44384	127.785	6/20/2024	
OK05	Japan	2	26.36446	127.876	6/22/2024	
OK06	Japan	2	26.71233	127.8813	6/21/2024	
OK07	Japan	2	26.61437	127.8937	6/21/2024	
OK08	Japan	2	26.35403	127.9973	6/23/2024	
OK09	Japan	2	26.3003	128.0041	6/23/2024	
OK10	Japan	2	26.3003	128.0041	6/22/2024	
