# Supplementary material for: A predictive framework for identifying source populations of non-native marine macroalgae: Chondria tumulosa in the Pacific Ocean
Source: PeerJ. 2025 Jun 23;13:e19610. doi: 10.7717/peerj.19610 (PMC12199741; doi:10.7717/peerj.19610)
Supplement: Supplemental Information 7 — Included are those generated in this study and those acquired from GenBank, sorted by BLAST similarity to NCBI GenBank accession NC057618, the published plastidial genome of the alga. Identities of specimens reflect the given GenBank name or the original identification of the specimen if generated in this study. [file peerj-13-19610-s007.rtf]

Identity	NCBI GenBank Accession	Lab Accession	Collection details	Similarity to Chondria tumulosa NC057618 (%)	Reference	
Chondria tumulosa	PV036782	ARS09882	Manawai (Pearl and Hermes Atoll), Hawai‘i, USA; 03.VIII.2019; coll. H. Spalding & T. Williams (AM19)	100	This study	
Chondria tumulosa	PV036783	ARS09883	Manawai (Pearl and Hermes Atoll), Hawai‘i, USA; 04.VIII.2019; coll. H. Spalding & T. Williams (AM26)	100	This study	
Chondria tumulosa	PV036784	ARS09884	Manawai (Pearl and Hermes Atoll), Hawai‘i, USA; 06.VIII.2019; coll. H. Spalding & T. Williams (AM60)	100	This study	
Chondria tumulosa	PV036785	ARS09885	Manawai (Pearl and Hermes Atoll), Hawai‘i, USA; 09.VIII.2019; coll. H. Spalding & T. Williams (AM88)	100	This study	
Chondria tumulosa	PV036786	ARS09888	Manawai (Pearl and Hermes Atoll), Hawai‘i, USA; 31.VII.2019; coll. H. Spalding & T. Williams (NWHI804a)	100	This study	
Chondria tumulosa	PV036801	ARS10955	Kuaihelani (Midway Atoll), Hawai‘i, USA; 12.VIII.2021; coll. H. Spalding & T. Williams	100	This study	
Chondrophycus cf. cartilagineus	PV036817	ARS11026	Pohnpei, Federated States of Micronesia; 08.VIII.1996; coll. K.J. McDermid (BISH682104)	97.38	This study	
Chondria sp.	PV036849	ARS11072	Gulf of Aqaba, Egypt; 24.V.1997; Coll. O. De Clerck (ODC581)	96.31	This study	
Chondria cf. hypoglossoides 	PV036826	ARS11042	Laing Island, Papua New Guinea; VI.1980; coll. E. Coppejans (HEC4449)	96.04	This study	
Chondria sp.	PV036840	ARS11060	Madang, Papua New Guinea; 30.VII.1990; coll. E. Coppejans & W. Prud'homme van Reine (Copp & PVR 13452)	96.04	This study	
Chondria sp.	PV036825	ARS11041	Laing Island, Papua New Guinea; VI.1980; coll. E. Coppejans (HEC4375)	95.91	This study	
Chondria dangeardii	HQ421169	ARS03592 	Kakahaia, Moloka‘i, Hawai‘i, USA; 21.III.2008; coll. K. Conklin	95.78	Sherwood et al. 2010	
Chondria sp.	PV036845	ARS11068	Eilat, Israel; 23.VI.2011; coll. T. Sauvage (HV03038)	95.78	This study	
Chondria sp.	PV036836	ARS11055	Krabi, Thailand; 11.IV.2007; coll. E. Coppejans, S. Pongparadon, & W. Chaitum (HEC16150A)	95.68	This study	
Chondria dangeardii	HQ421190	ARS03726 	Ma‘alaea, Maui, Hawai‘i, USA; 6.IV.2008; coll. A. Kurihara	95.65	Sherwood et al. 2010	
Chondria sp.	PV036827	ARS11043	Laing Island, Papua New Guinea; VII.1980; coll. E. Coppejans (HEC4526)	95.65	This study	
Chondria sp.	PV036828	ARS11044	Laing Island, Papua New Guinea; VII.1980; coll. E. Coppejans (HEC4556)	95.65	This study	
Chondria sp.	PV036842	ARS11064	Madang, Papua New Guinea; 22.VIII.1990; coll. E. Coppejans & W. Prud'homme van Reine (Copp & PVR 13795)	95.38	This study	
Chondria cf. hypoglossoides 	PV036824	ARS11040	Laing Island, Papua New Guinea; VI.1980; coll. E. Coppejans (HEC4363)	95.32	This study	
Chondria sp.	HQ421167	ARS03588 	Moloka‘i, Hawai‘i, USA; 21.III.2008; coll. K. Conklin	95.25	Sherwood et al. 2010	
Chondria sp.	PV036829	ARS11045	Laing Island, Papua New Guinea; VIII.1980; coll. E. Coppejans (HEC4702)	95.25	This study	
Chondria cf. dangeardii	PV036833	ARS11052	Cape Iris, Papua New Guinea; 26.VII.1988; coll. E. Coppejans (HEC7982)	95.25	This study	
Chondria sp.	PV036835	ARS11054	Diani, Kenya; 24.VIII.1989; coll. E. Coppejans (HEC8498)	95.25	This study	
Chondria sp.	PV036847	ARS11070	Rhodes, Greece; 10.IX.2011; coll. H. Verbruggen, F. Leliaert, & O. De Clerck (HV03283)	95.25	This study	
Chondria cf. dangeardii 	PV036848	ARS11071	Masirah, Oman; 19.XI.1999; coll. T. Schils (MAS533)	95.19	This study	
Chondria sp.	OM468962	BLB_1373	Kāneʻohe, Hawai‘i, USA; 23.V.2017; coll. B. Brooks	95.14	MarineGEO	
Chondria sp.	PV036832	ARS11050	Jais Aben, Papua New Guinea; 20.VI.1988; coll. E. Coppejans (HEC7515)	95.12	This study	
Chondria sp.	PV036843	ARS11065	Madang, Papua New Guinea; 22.VIII.1990; coll. E. Coppejans & W. Prud'homme van Reine (Copp & PVR 13796)	95.12	This study	
Chondria sp.	PV036846	ARS11069	Rhodes, Greece; 7.IX.2011; coll. H. Verbruggen & O. De Clerck (HV03115)	95.12	This study	
Chondria sp.	PP862947	MARI-04556	Rhode Island, USA; 1.VIII.2023; coll. T. Irvine	95.12	Irvine, Wysor, and Beauvais 2024	
Polysiphonia sp.	KY573920	PHY4526	Punta Biruca, Panama; 08.I.2011; coll. B. Wysor & D.W. Freshwater	95.12	Freshwater et al. 2017	
Chondria sp.	HQ421003	ARS02062 	Paiko, O‘ahu, Hawai‘i, USA; 22.XII.2006; coll. A. Kurihara	95.11	Sherwood et al. 2010	
Chondria sp.	PV036841	ARS11063	Madang, Papua New Guinea; 22.VIII.1990; coll. E. Coppejans & W. Prud'homme van Reine (Copp & PVR 13792)	95.05	This study	
Chondria sp.	HQ420915	ARS01780	O‘ahu; Hawai‘i, USA; 15.XII.2006; coll. H. Spalding	94.99	Sherwood et al. 2010	
Chondria sp.	HQ421558	ARS01783 	O‘ahu; Hawai‘i, USA; 15.XII.2006; coll. H. Spalding	94.99	Sherwood et al. 2010	
Chondria cf. armata 	PV036830	ARS11046	Laing Island, Papua New Guinea; VIII.1986; coll. E. Coppejans (HEC6466)	94.99	This study	
Chondria sp.	PV036839	ARS11058	Ulingan Bay, Madang Province, Papua New Guinea; 21.VII.1990; coll. E. Coppejans & W. Prud'homme van Reine (Copp & PVR 13306)	94.99	This study	
Osmundea sinicola	NC_51457	UC2041541 	Baja California, Mexico; – ; coll. Marchant	94.99	Hughey and Miller 2021	
Chondria atropurpurea	PP862937	MARI-04465	Rhode Island, USA; 21.VI.2023; coll. T. Irvine	94.85	Irvine, Wysor, and Beauvais 2024	
Ululania stellata	HQ421198	ARS03719 	Punalu‘u, Hawai‘i Island, Hawai‘i, USA; 24.I.2008; coll. A. Kurihara	94.84	Sherwood et al. 2010	
Laurencia nidifica	HQ420935	ARS00849 	Lipoa, Maui, Hawai‘i, USA; 5.IV.2006; coll. A. Carlile	94.72	Sherwood et al. 2010	
Herposiphonia sp. 	HQ421121	ARS03412 	Mahukona, Hawai‘i Island, Hawai‘i, USA; 21.II.2008; coll. K. Conklin	94.72	Sherwood et al. 2010	
Chondria cf. atropurpurea	PV036788	ARS10455	St. Croix, US Virgin Islands; 5.I.1977; coll. I.A. Abbott (BISH 693107)	94.72	This study	
Laurencia sp.	PV036791	ARS10462	Chonburi, Thailand; 12.I.2019; coll. S. Draisma (SGAD1901036)	94.72	This study	
Chondria sp.	PV036792	ARS10463	Maitai Bay, Aotearoa (New Zealand); 23.I.2015; coll. W. Nelson (ASP016)	94.72	This study	
Chondria cf. macrocarpa	PV036795	ARS10468	Oneroa Bay, Aotearoa (New Zealand); 29.11.2019; coll. R. D’Archino & W. Nelson (AST376; WELT AK379909)	94.72	This study	
Chondria cf. macrocarpa	PV036796	ARS10471	Moa Point, Aotearoa (New Zealand); 1.XI.2021; coll. R. D’Archino (ASV017)	94.72	This study	
Chondrophycus cf. undulatus 	PV036816	ARS11022	Hinatunglan, Philippines; 15.VI.1969; coll. G.A. Santos (BISH536254)	94.72	This study	
Chondria cf. armata 	PV036820	ARS11034	Weligama, Sri Lanka; 10.I.1997; coll. E. Coppejans (HEC11634)	94.72	This study	
Chondria sp.	PV036851	ARS11192	São Paulo, Brazil; 14.V.2010; coll. M.T. Fujii (IBT0359)	94.72	This study	
Laurenciella marilzae	NC_35259	HV1501 	Pta Hidalgo, Tenerife, Spain; 10.VI.2015; –	94.72	Díaz-Tapia et al. 2017	
Laurencieae 	MF101412	JFC1711 	Margate, KwaZulu-Natal, South Africa; 12.XII.2014; –	94.72	Díaz-Tapia et al. 2017	
Chondria sp.	MF101429	PD620 	Praia de Parati, Espírito Santo, Brazil; 8.IV.2014; –	94.72	Díaz-Tapia et al. 2017	
Chondria sp.	MF101431	PD745 	Port Arlington, Victoria, Australia; 9.XI.2014; –	94.72	Díaz-Tapia et al. 2017	
Chondria baileyana	PP862944	MARI-04545	Massachusetts, USA; 29.VII.2023; coll. T. Irvine	94.58	Irvine, Wysor, and Beauvais 2024	
Chondrophycus cartilagineus	HQ421428	ARS00687 	Laniupoko, Maui, Hawai‘i, USA; 8.II.2001; coll. C.F. Puttock (BISH668929)	94.57	Sherwood et al. 2010	
Laurencia sp.	HQ420951	ARS02799 	Lalo (French Frigate Shoals), Hawai‘i, USA; 21.XI.2006; coll. R. Most	94.46	Sherwood et al. 2010	
Laurencia sp.	HQ421168	ARS03589 	Moloka‘i, Hawai‘i, USA; 21.III.2008; coll. K. Conklin	94.46	Sherwood et al. 2010	
Chondria cf. armata 	PV036822	ARS11036	Dickwella, Sri Lanka; 23.I.2007; coll. E. Coppejans (HEC16029)	94.46	This study	
Chondria cf. armata 	PV036823	ARS11037	Confifi, Sri Lanka; 26.I.2007; coll. E. Coppejans (HEC16071)	94.46	This study	
Amplisiphonia pacifica	NC_70145	GWS020992 	Haida Gwaii, British Columbia, Canada; 8.VI.2017; coll. G.W. Saunders & K. Dixon	94.46	Díaz-Tapia et al. 2022	
Herposiphonia akidoglossa	MT939887	Heak-2020-1	–	94.46	Kundu 2022	
Laurencia sp.	LN833431	JFC0032 	Coral Bay, Australia; – ; coll. M. Van Keulen & F. McGregor	94.46	Verbruggen and Costa 2015	
Lophurella hookeriana	NC_79642	Y1 	Yendegaia, Patagonia, Chile; 22.X.2016; –	94.46	Díaz-Tapia, Nelson, and Verbruggen 2023	
Chondrophycus cartilagineus	HQ421531	ARS00733 	Wailupe, O‘ahu, Hawai‘i, USA; 27.III.1994; coll. L.M. Hodgson (BISH663223)	94.33	Sherwood et al. 2010	
Laurencia sp.	PV036808	ARS10989	Katirirake, Kiribati; 18.VI.1970; coll. G. Groves (BISH535635)	94.33	This study	
Ululania stellata	HQ421124	ARS03567 	Kūpikipikiʻō (Black Point), O‘ahu, Hawai‘i, USA; 11.III.2008; coll. A. Kurihara	94.29	Sherwood et al. 2010	
Laurencia sp.	HQ421371	ARS04513 	Kailua, O‘ahu, Hawai‘i, USA; 7.II.2009; coll. A. Kurihara	94.29	Sherwood et al. 2010	
Acanthophora spicifera	EF426609	ARS00126	Hunakai, Oahu, Hawai‘i, USA; 16.I.2005; –	94.2	Sherwood and Presting 2007	
Laurencia sp.	HQ421537	ARS00785 	Kaua‘i, Hawai‘i, USA; 5.III.2005; coll. A.R. Sherwood & G. Presting	94.2	Sherwood et al. 2010	
Herposiphonia sp.	HQ421141	ARS03084 	Lualualei, O‘ahu, Hawai‘i, USA; 12.XI.2007; coll. A. Kurihara	94.2	Sherwood et al. 2010	
Acanthophora spicifera	HQ421079	ARS03203 	Hilo, Hawai‘i Island, Hawai‘i, USA; 25.I.2008; coll. A. Kurihara & K. Conklin	94.2	Sherwood et al. 2010	
Chondria cf. lanceolata 	PV036793	ARS10465	Urupukapuka Island, Aotearoa (New Zealand); 8.IV.2006; coll. W. Nelson (ASG732)	94.2	This study	
Laurencia sp.	PV036799	ARS10495	Plaja Kanoa, Curaçao; 29.III.2015; coll. S. Draisma (SGAD1502186)	94.2	This study	
Chondria sp.	PV036831	ARS11048	Melindi, Kenya; 19.III.1988; coll. E. Coppejans (HEC7391)	94.2	This study	
Echinothamnion sp.	OQ731395	ASR228 	Kaikoura, Aotearoa (New Zealand); 15.X.2017; –	94.2	Díaz-Tapia, Nelson, and Verbruggen 2023	
Lophurella mutabilis	NC_79643	ASR85 	Stewart Island, Aotearoa (New Zealand); 1.IV.2017; –	94.2	Díaz-Tapia, Nelson, and Verbruggen 2023	
Periphykon beckeri	NC_35261	JH1427 	Barrow Island, Western Australia; 16.XI.2014; –	94.2	Díaz-Tapia et al. 2017	
Lophurella sp.	OQ731399	PD2877 	Shipwreck Creek, Mallacoota, Victoria, Australia; 13.XI.2016; coll P. Díaz-Tapia	94.2	Díaz-Tapia, Nelson, and Verbruggen 2023	
Echinothamnion hystrix	NC_79647	PD3279 	Wynyard, Tasmania, Australia; 4.XI.2017; coll. P. Díaz-Tapia & J. Costa	94.2	Díaz-Tapia, Nelson, and Verbruggen 2023	
Laurencia catarinensis	NC_82854	PD4141 	Charco de la Novia, Lanzarote, Canary Islands, Spain; 11.VI.2018; – 	94.2	Preuss et al. 2023	
Chondria sp.	PV036797	ARS10491	Puerto Galera, Philippines; 28.XI.2013; coll. S. Draisma (SGAD1312034)	94.08	This study	
Laurencia sp. 	PV036809	ARS10990	Abemama, Kiribati; 18.VI.1970; coll. G. Groves (BISH535687)	94.08	This study	
Chondrophycus sp.	HQ421454	ARS01994 	Maui, Hawai‘i, USA; 27.I.2007; coll. T. Shipley	94.06	Sherwood et al. 2010	
Chondria sp.	PV036794	ARS10467	Tapeka Point (Bay of Islands), Aotearoa (New Zealand); 29.IV.2010; coll. W. Nelson (ASK116; WELT A030650; WELT A030649)	94.06	This study	
Laurencia elata	NC_82855	B2H 	Barwon Heads, Victoria, Australia; 11.VI.2021; –	94.06	Preuss et al. 2023	
Lophocladia sp.	HQ421288	ARS00665 	Ala Moana, O‘ahu, Hawai‘i, USA; 2.II.2006; coll. M. Ross	93.93	Sherwood et al. 2010	
Laurencia majuscula	HQ421529	ARS00686 	Hanauma Bay, O‘ahu, Hawai‘i, USA; 9.V.2000; coll. R. Okano (BISH691443)	93.93	Sherwood et al. 2010	
Lophocladia sp.	EF426608	ARS00772	Kawaihoa, O‘ahu, Hawai‘i, USA; 27.XII.2005; coll. M. Ross	93.93	Sherwood and Presting 2007	
Laurencia majuscula	HQ420941	ARS01603 	Lalo (French Frigate Shoals), Hawai‘i, USA; XI.2006; coll. R. Most 	93.93	Sherwood et al. 2010	
Acanthophora pacifica	HQ421463	ARS02010 	Ma‘alaea, Maui, Hawai‘i, USA; 28.I.2007; coll. K. Conklin	93.93	Sherwood et al. 2010	
Laurencia majuscula	HQ421515	ARS02316 	Anahola, Kaua‘i, Hawai‘i, USA; 17.III.2007; coll. K. Conklin	93.93	Sherwood et al. 2010	
Laurencia sp.	HQ421516	ARS02317 	Anahola, Kaua‘i, Hawai‘i, USA; 17.III.2007; coll. K. Conklin	93.93	Sherwood et al. 2010	
Laurencia majuscula	HQ421517	ARS02321 	Lawai Beach, Kaua‘i, Hawai‘i, USA; 18.III.2007; coll. K. Conklin	93.93	Sherwood et al. 2010	
Laurencia majuscula	HQ421514	ARS02332 	Anini, Kaua‘i, Hawai‘i, USA; 17.III.2007; coll. K. Conklin	93.93	Sherwood et al. 2010	
Acanthophora pacifica	HQ421072	ARS03118 	Hamoa, Maui, Hawai‘i, USA; 11.XII.2007; coll. K. Conklin	93.93	Sherwood et al. 2010	
Laurencia sp.	HQ421088	ARS03166 	Honomanu, Maui, Hawai‘i, USA; 31.I.2008; coll. S. Hau	93.93	Sherwood et al. 2010	
Chondria sp.	PV036800	ARS10496	Bali, Indonesia; 19.VI.2015; Coll. S. Draisma (SGAD1506067)	93.93	This study	
Laurencia sp.	PV036812	ARS10993	Ko Ton, Thailand; 26.I.1995; coll. M.D. Hoyle (BISH639215)	93.93	This study	
Chondria floridana 	PV036850	ARS11184	Santa Catarina, Brazil; 31.III.2006; coll. M. Noseda & M.T. Fujii (IBT592)	93.93	This study	
Laurencia australis	OQ908868	B1P 	Victoria, Australia; – ; –	93.93	Preuss et al. 2023	
Laurencia australis	OQ908869	B2P 	Victoria, Australia; – ; –	93.93	Preuss et al. 2023	
Chondria sp.	MF101451	PD1582 	Rottnest Island, Western Australia; 15.III.2015; –	93.93	Díaz-Tapia et al. 2017	
Polysiphonia sp.	MF101456	PD1760 	Coral Bay, Western Australia; 24.III.2015; –	93.93	Díaz-Tapia et al. 2017	
Echinothamnion hookeri	NC_79646	PD3033	George Town, Tasmania, Australia; 5.XI.2017; coll. P. Díaz-Tapia & J. Costa	93.93	Díaz-Tapia, Nelson, and Verbruggen 2023	
Tayloriella tenebrosa	NC_70147	PD4020 	Hermanaus, South Africa; – ; coll. P. Díaz-Tapia	93.93	Díaz-Tapia et al. 2022	
Laurencia obtusa	KC795895	UQDHQW20121115	Qingdao, China; XI.2012; coll. G. Du	93.93	Du et al. 2015	
Laurencia cf. nipponica 	PV036815	ARS11020	Osyoro, Japan; 24.VI.1945; coll. T. Segi (BISH535734)	93.8	This study	
Lophocladia sp.	HQ421285	ARS00548 	Portlock, O‘ahu; Hawai‘i, USA; 1.VII.2005; coll. M. Ross	93.67	Sherwood et al. 2010	
Lophocladia sp.	EF426606	ARS00771	Lānaʻi Lookout, O‘ahu, Hawai‘i, USA; 27.XII.2005; coll. M. Ross	93.67	Sherwood and Presting 2007	
Lophocladia sp.	EF426607	ARS00773	Kawaihoa, O‘ahu, Hawai‘i, USA; 27.XII.2005; coll. M. Ross	93.67	Sherwood and Presting 2007	
Lophocladia sp.	HQ421641	ARS01590 	Hawai‘i, USA; XII.2006; coll. H. Spalding (HS465)	93.67	Sherwood et al. 2010	
Laurencia mcdermidiae	HQ421155	ARS03611 	Kanalukaha, Moloka‘i, Hawai‘i, USA; 23.III.2008; coll. K. Conklin	93.67	Sherwood et al. 2010	
Chondria sp. 	PV036807	ARS10987	Matuku, Fiji; 1.IX.1968; coll. Y. Kondo (BISH528582)	93.67	This study	
Laurencia sp.	PV036814	ARS11018	Ono-i-Lau, Fiji; VIII.1968; coll. Y. Kondo (BISH535555)	93.67	This study	
Chondria sp.	PV036852	ARS11201	Espírito Santo, Brazil; 12.XI.2012; coll. M.T. Fujii (MTF23)	93.67	This study	
Rhodomelopsis africana	NC_70142	GWS036468 	Shelley Beach, South Africa; 8.XII.2014; coll. K. Dixon & J. Costa 	93.67	Díaz-Tapia et al. 2022	
Lophocladia kuetzingii	NC_35292	PD1509 	Albany, Western Australia; 11.III.2015; –	93.67	Díaz-Tapia et al. 2017	
Symphyocladia marchantioides	NC_70148	PD3704 	Praia do Populo, Azores, Portugal; 12.IV.2018; coll. P. Díaz-Tapia	93.67	Díaz-Tapia et al. 2022	
Laurencia verruciformis	OQ908870	PD4142 	Charco de la Novia, Lanzarote, Canary Islands, Spain; 3.IV.2021; –	93.67	Preuss et al. 2023	
Chondria sp.		ARS11067	Eilat, Israel; 23.VI.2011; coll. T. Sauvage (HV03037)	93.62	This study	
Palisada intermedia	NC_82175	AF489863	Taiping, Republic of China (Taiwan); 20.XI.2017; coll. P. Chen 	93.4	Chen, Perez, & Liu 2022	
Spirocladia barodensis	HQ421562	ARS02022 	Honolua, Maui, Hawai‘i, USA; 27.II.2007; coll. T. Sauvage	93.4	Sherwood et al. 2010	
Palisada parvipapillata	HQ421459	ARS02288 	Moloka‘i, Hawai‘i, USA; 11.II.2007; coll. T. Shipley	93.4	Sherwood et al. 2010	
Herposiphonia sp.	HQ421140	ARS03083 	Lualualei, O‘ahu, Hawai‘i, USA; 12.XI.2007; coll. A. Kurihara	93.4	Sherwood et al. 2010	
Palisada parvipapillata	HQ421146	ARS03381 	Puako, Hawai‘i Island, Hawai‘i, USA; 21.II.2008; coll. K. Conklin	93.4	Sherwood et al. 2010	
Chondrophycus cf. succisus 	PV036818	ARS11027	Pohnpei, Federated States of Micronesia; 24.XI.1994; coll. L.M. Hodgson (BISH682106)	93.4	This study	
Chondrophycus cf. dotyi 	PV036819	ARS11029	Lisianski, Hawai‘i, USA; 22.IX.2000; coll. K.J. Geisler (BISH698678)	93.4	This study	
Lophurella caespitosa	NC_79641	ASR202 	Arthur’s Nose, Wellington, Aotearoa (New Zealand); 21.VI.2017; coll. W. Nelson 	93.4	Díaz-Tapia, Nelson, and Verbruggen 2023	
Polysiphonia sp.	MF101414	JH1432 	Barrow Island, Western Australia; 14.XI.2014; –	93.4	Díaz-Tapia et al. 2017	
Thaumatella adunca	NC_35291	PD1388 	The Rip, Victoria, Australia; 29.I.2015; –	93.4	Díaz-Tapia et al. 2017	
Palisada sp.	MF101453	PD1686 	Two Rocks, Western Australia; 19.III.2015; –	93.4	Díaz-Tapia et al. 2017	
Deltalsia parasitica	NC_70143	PD2962	Skomer Island, Wales, UK; 11.VIII.2016; –	93.4	Díaz-Tapia et al. 2022	
Laurencia obtusa	NC_82856	PD4197 	El Golfo, Lanzarote, Canary Islands, Spain; 11.VI.2018; –	93.4	Preuss et al. 2023	
Pterosiphonia complanata	NC_70144	PD4865 	Ártabra, A Coruña, Spain; 5.VII.2019; coll. P. Díaz-Tapia	93.4	Díaz-Tapia et al. 2022	
Xiphosiphonia pinnulata	NC_70146	PD4937 	Bastiagueiro, A Coruña, Spain; 01.X.2019; coll. P. Díaz-Tapia	93.4	Díaz-Tapia et al. 2022	
Symphyocladia latiuscula	KC795875	UQDMTJ20111126	Qingdao, China; XI.2011; coll. G. Du	93.4	Du et al. 2015	
Spyridia filamentosa	HQ420959	ARS02812 	Paiko, O‘ahu, Hawai‘i, USA; 10.IV.2006; coll. K. Conklin	93.27	Sherwood et al. 2010	
Chondria sp.	PV036805	ARS10985	Florida, USA; 30.VII.1968; coll. M.D. Hoyle, (BISH528555)	93.27	This study	
Ceramiales	PV036790	ARS10459	Calamian Is, Philippines; 3.VII.2014; coll. S. Draisma (SGAD1403020 BUS1)	93.14	This study	
Chondria sp.	PV036834	ARS11053	Chale Island, Kenya; 9.VIII.1989; coll. E. Coppejans (HEC8314)	93.14	This study	
Chondria sp.	PV036838	ARS11057	Burgas, Bulgaria; 7.VI.2011; coll. O. De Clerck, F. Steen (FS1070)	93.14	This study	
Chondria cf. nudifica	PV036787	ARS10441	Goleta, California, USA; 27.VIII.1965; coll. M. Neushul (BISH 528692)	93.07	This study	
Chondrophycus cf. surculigerus	PV036813	ARS11013	Laysan, Hawai‘i, USA; 6.XII.1963; coll. R.T. Tsuda (BISH518323)	93.03	This study	
Chondria sp. 	PV036806	ARS10986	Ono-i-Lau, Fiji; 1.VIII.1968; coll. Y. Kondo (BISH528580)	92.88	This study	
Acanthophora cf. spicifera	PV036804	ARS10984	Tarawa, Kiribati; 1.II.1974; coll. Hyde (BISH526053)	92.7	This study	
Chondria cf. ryukuensis	PV036789	ARS10457	Johor, Malaysia; 22.VI.2012; coll. S. Draisma (SGAD1205064)	92.48	This study	
Acanthophora sp.	PV036803	ARS10982	Samar, Philippines; 17.VI.1969; coll. G.A. Santos (BISH525801)	92.43	This study	
Laurencia sp.	PV036811	ARS10992	Hainan, China; 4.XII.1981; coll. M.S. Doty (BISH621762)	92.15	This study	
Chondria sp.	PV036837	ARS11056	Chilaw, Sri Lanka; 29.IX.2007; coll. E. Coppejans (HEC16280)	91.29	This study	
Chondria sp.	PV036798	ARS10492	Calamian Is., Philippines; 6.III.2014; coll. S. Draisma (SGAD1403119)	90.53	This study	
Chondria sp.	PV036821	ARS11035	Porto da Cruz, Portugal; 16.V.2006; coll. E. Coppejans & O. De Clerck (HEC15797)	90.38	This study	
Laurencia sp. 	PV036810	ARS10991	Abemama, Kiribati; 18.VI.1970; coll. G. Groves (BISH535688)	90.37	This study	
Chondria cf. dasyphylla	PV036802	ARS10981	Bushire, Iran; 12.VIII.1937; coll. M. Køie (BISH520753)	88.75	This study	
