# Supplementary material for: A predictive framework for identifying source populations of non-native marine macroalgae: Chondria tumulosa in the Pacific Ocean
Source: PeerJ. 2025 Jun 23;13:e19610. doi: 10.7717/peerj.19610 (PMC12199741; doi:10.7717/peerj.19610)
Supplement: Supplemental Information 8 — The species column includes those that are either currently accepted taxonomically, have unresolved taxonomic status, or have been transferred to other genera within the family Rhodomelaceae. Availability of DNA barcode sequences for these species from GenBank NCBI is indicated. [file peerj-13-19610-s008.rtf]

Species	Japan	Line Islands including Palmyra	Johnston Atoll	Clipperton	Galápagos	Available NCBI GenBank sequences	
Acanthophora muscoides	X					None	
Acanthophora spicifera	X					18S, 28S, COI, LSU, petB, psbA, rbcL, SSU, UPA	
Chondria acrorhizophora					X	None	
Chondria armata	X					COI, rbcL, SSU	
Chondria capillaris	X					rbcL, SSU	
Chondria clarionensis				X		None	
Chondria crassicaulis	X					18S, COI, LSU, SSU, rbcL	
Chondria dasyphylla	X					18S, 28S, COI, LSU, petB, psbA, rbcL, SSU, UPA	
Chondria econstricta	X					None	
Chondria expansa	X					18S, COI, SSU, rbcL	
Chondria flexicaulis					X	None	
Chondria intertexta	X					rbcL	
Chondria lancifolia	X					None	
Chondria mageshimensis	X					rbcL	
Chondria minitula		X				None	
Chondria polyrhiza	X		X			rbcL	
Chondria repens	X	X				None	
Chondria ryukuensis	X					COI, SSU, rbcL 	
Chondria simpliciuscula		X	X			None	
Chondria stolonifera	X					None	
Chondria xishaensis	X					None	
Laurencia filiformis						18S, SSU, rbcL	
Laurencia pinnata	X					None	
Palisada perforata	X			X		COI, psbA, rbcL 	
