# Supplementary material for: A predictive framework for identifying source populations of non-native marine macroalgae: Chondria tumulosa in the Pacific Ocean
Source: PeerJ. 2025 Jun 23;13:e19610. doi: 10.7717/peerj.19610 (PMC12199741; doi:10.7717/peerj.19610)
Supplement: Supplemental Information 9 — Longitude and latitude represent the centroid of each polygon. Latitude is given in positive and negative values, with those below zero representing degrees south. Longitude is given from 0-360 with those values of western longitude represented by positive numbers. This was necessary for modeling the particles across the meridian. [file peerj-13-19610-s009.rtf]

Polygon ID	landings	long	lat	
1	0	158.24	-18.63	
2	0	158.24	-19.63	
3	0	158.24	-20.63	
4	0	158.24	-21.63	
5	0	159.11	-19.13	
6	0	159.11	-20.13	
7	0	159.11	-21.13	
8	0	159.11	-22.13	
9	0	159.98	-21.63	
10	0	162.57	-18.13	
11	0	162.57	-19.13	
12	0	163.44	-17.63	
13	0	163.44	-18.63	
14	0	163.44	-19.63	
15	0	163.44	-20.63	
16	0	164.31	-18.13	
17	0	164.31	-19.13	
18	0	164.31	-20.13	
19	0	164.31	-21.13	
20	0	165.17	-19.63	
21	0	165.17	-20.63	
22	0	165.17	-21.63	
23	0	166.04	-19.13	
24	0	166.04	-20.13	
25	0	166.04	-21.13	
26	0	166.04	-22.13	
27	0	166.04	-23.13	
28	0	166.91	-20.63	
29	0	166.91	-21.63	
30	0	166.91	-22.63	
31	0	166.91	-23.63	
32	0	167.77	-21.13	
33	0	167.77	-22.13	
34	0	167.77	-23.13	
35	0	168.64	-21.63	
36	0	168.64	-22.63	
37	0	166.04	-13.13	
38	0	166.04	-14.13	
39	0	166.04	-15.13	
40	0	166.91	-12.63	
41	0	166.91	-13.63	
42	0	166.91	-14.63	
43	0	166.91	-15.63	
44	0	166.91	-16.63	
45	0	167.77	-13.13	
46	0	167.77	-14.13	
47	0	167.77	-15.13	
48	0	167.77	-16.13	
49	0	167.77	-17.13	
50	0	167.77	-18.13	
51	0	168.64	-14.63	
52	0	168.64	-15.63	
53	0	168.64	-16.63	
54	0	168.64	-17.63	
55	0	168.64	-18.63	
56	0	168.64	-19.63	
57	0	169.5	-18.13	
58	0	169.5	-19.13	
59	0	169.5	-20.13	
60	0	170.37	-19.63	
61	0	170.37	-20.63	
62	0	154.78	-7.63	
63	0	155.65	-6.13	
64	0	155.65	-7.13	
65	0	155.65	-8.13	
66	0	156.51	-6.63	
67	0	156.51	-7.63	
68	0	156.51	-8.63	
69	0	157.38	-7.13	
70	0	157.38	-9.13	
71	0	158.24	-6.63	
72	0	158.24	-7.63	
73	0	158.24	-8.63	
74	0	158.24	-9.63	
75	0	159.11	-5.13	
76	0	159.11	-6.13	
77	0	159.11	-7.13	
78	0	159.11	-8.13	
79	0	159.11	-9.13	
80	0	159.11	-10.13	
81	0	159.11	-11.13	
82	0	159.98	-5.63	
83	0	159.98	-7.63	
84	0	159.98	-9.63	
85	0	159.98	-10.63	
86	0	159.98	-11.63	
87	0	159.98	-12.63	
88	0	160.84	-8.13	
89	0	160.84	-9.13	
90	0	160.84	-10.13	
91	0	160.84	-11.13	
92	0	160.84	-12.13	
93	0	160.84	-13.13	
94	0	161.71	-8.63	
95	0	161.71	-9.63	
96	0	161.71	-10.63	
97	0	162.57	-8.13	
98	0	162.57	-9.13	
99	0	162.57	-10.13	
100	0	162.57	-11.13	
101	0	163.44	-8.63	
102	0	165.17	-9.63	
103	0	165.17	-10.63	
104	0	166.04	-10.13	
105	0	166.04	-11.13	
106	0	166.04	-12.13	
107	0	166.91	-9.63	
108	0	166.91	-10.63	
109	0	166.91	-11.63	
110	0	167.77	-10.13	
111	0	168.64	-11.63	
112	0	168.64	-12.63	
113	0	169.5	-11.13	
114	0	169.5	-12.13	
115	0	170.37	-11.63	
116	0	130.53	3.37	
117	0	131.4	4.87	
118	0	131.4	3.87	
119	0	131.4	2.87	
120	0	132.26	6.37	
121	0	132.26	5.37	
122	0	132.26	4.37	
123	0	132.26	3.37	
124	0	133.13	6.87	
125	0	133.13	5.87	
126	0	133.13	4.87	
127	11	133.13	3.87	
128	0	134	8.37	
129	0	134	7.37	
130	0	134	6.37	
131	0	134	5.37	
132	0	134.86	8.87	
133	0	134.86	7.87	
134	0	134.86	6.87	
135	0	137.46	9.37	
136	0	137.46	8.37	
137	0	138.33	9.87	
138	0	138.33	8.87	
139	1	139.19	10.37	
140	0	139.19	9.37	
141	0	140.06	9.87	
142	0	140.06	7.87	
143	0	140.92	9.37	
144	0	140.92	8.37	
145	0	142.66	7.37	
146	0	142.66	6.37	
147	0	143.52	7.87	
148	0	143.52	6.87	
149	0	144.39	8.37	
150	0	144.39	7.37	
151	1	145.25	9.87	
152	0	145.25	8.87	
153	0	145.25	7.87	
154	0	146.12	8.37	
155	0	146.12	7.37	
156	0	146.99	7.87	
157	0	146.99	6.87	
158	1	147.85	8.37	
159	0	148.72	7.87	
160	0	148.72	6.87	
161	3	149.58	9.37	
162	1	149.58	8.37	
163	0	149.58	7.37	
164	0	149.58	6.37	
165	0	150.45	8.87	
166	0	150.45	7.87	
167	0	151.32	8.37	
168	0	151.32	7.37	
169	5	152.18	8.87	
170	0	152.18	7.87	
171	0	152.18	6.87	
172	0	153.05	7.37	
173	0	153.05	6.37	
174	0	153.05	5.37	
175	0	153.91	7.87	
176	0	153.91	5.87	
177	0	153.91	4.87	
178	3	154.78	8.37	
179	0	154.78	7.37	
180	0	154.78	4.37	
181	20	154.78	3.37	
182	2	154.78	1.37	
183	3	154.78	0.37	
184	3	155.65	7.87	
185	1	155.65	6.87	
186	26	155.65	3.87	
187	1	156.51	5.37	
188	3	157.38	6.87	
189	0	157.38	5.87	
190	14	158.24	7.37	
191	0	158.24	6.37	
192	1	159.11	6.87	
193	14	159.98	7.37	
194	0	159.98	6.37	
195	1	160.84	6.87	
196	0	160.84	5.87	
197	5	162.57	5.87	
198	3	162.57	4.87	
199	4	163.44	5.37	
200	9	166.91	-0.63	
201	0	116.68	8.37	
202	0	116.68	7.37	
203	0	117.54	9.87	
204	0	117.54	8.87	
205	0	117.54	7.87	
206	0	117.54	6.87	
207	0	118.41	10.37	
208	0	118.41	9.37	
209	0	118.41	7.37	
210	0	118.41	6.37	
211	0	118.41	5.37	
212	0	119.27	16.87	
213	0	119.27	15.87	
214	0	119.27	14.87	
215	0	119.27	13.87	
216	0	119.27	12.87	
217	0	119.27	11.87	
218	0	119.27	10.87	
219	0	119.27	5.87	
220	0	119.27	4.87	
221	0	119.27	3.87	
222	0	120.14	18.37	
223	0	120.14	17.37	
224	0	120.14	16.37	
225	0	120.14	15.37	
226	0	120.14	14.37	
227	0	120.14	13.37	
228	0	120.14	12.37	
229	0	120.14	9.37	
230	0	120.14	8.37	
231	0	120.14	5.37	
232	0	120.14	4.37	
233	0	121.01	20.87	
234	0	121.01	19.87	
235	0	121.01	18.87	
236	0	121.01	8.87	
237	0	121.01	5.87	
238	0	121.01	4.87	
239	0	121.87	21.37	
240	0	121.87	20.37	
241	0	121.87	19.37	
242	0	121.87	18.37	
243	0	121.87	17.37	
244	0	121.87	16.37	
245	0	121.87	6.37	
246	0	121.87	5.37	
247	0	122.74	19.87	
248	0	122.74	18.87	
249	0	122.74	17.87	
250	0	122.74	16.87	
251	0	122.74	15.87	
252	0	122.74	14.87	
253	0	122.74	6.87	
254	0	122.74	5.87	
255	0	123.6	14.37	
256	0	123.6	6.37	
257	0	124.47	13.87	
258	0	124.47	12.87	
259	0	124.47	5.87	
260	0	125.34	13.37	
261	0	125.34	12.37	
262	0	125.34	11.37	
263	0	125.34	5.37	
264	0	126.2	11.87	
265	0	126.2	10.87	
266	0	126.2	9.87	
267	0	126.2	8.87	
268	0	126.2	7.87	
269	0	126.2	6.87	
270	0	126.2	5.87	
271	0	127.07	8.37	
272	0	127.07	7.37	
273	0	140.92	-2.63	
274	0	140.92	-9.63	
275	0	141.79	-2.13	
276	0	141.79	-3.13	
277	0	141.79	-9.13	
278	0	142.66	-1.63	
279	0	142.66	-9.63	
280	0	143.52	-1.13	
281	0	143.52	-8.13	
282	0	143.52	-9.13	
283	0	144.39	-0.63	
284	0	144.39	-1.63	
285	0	144.39	-7.63	
286	0	144.39	-8.63	
287	0	145.25	-0.13	
288	0	145.25	-1.13	
289	0	145.25	-8.13	
290	0	146.12	-0.63	
291	0	146.12	-1.63	
292	0	146.12	-8.63	
293	0	146.12	-9.63	
294	0	146.99	-2.13	
295	0	146.99	-9.13	
296	0	146.99	-10.13	
297	0	147.85	-1.63	
298	0	147.85	-9.63	
299	0	147.85	-10.63	
300	0	148.72	-1.13	
301	0	148.72	-2.13	
302	0	148.72	-10.13	
303	0	148.72	-11.13	
304	0	149.58	-0.63	
305	0	149.58	-1.63	
306	0	149.58	-10.63	
307	0	150.45	-1.13	
308	0	150.45	-2.13	
309	0	150.45	-6.13	
310	0	150.45	-7.13	
311	0	150.45	-8.13	
312	0	150.45	-11.13	
313	0	151.32	-1.63	
314	0	151.32	-2.63	
315	0	151.32	-5.63	
316	0	151.32	-6.63	
317	0	151.32	-7.63	
318	0	151.32	-8.63	
319	0	151.32	-10.63	
320	0	151.32	-11.63	
321	1	152.18	-2.13	
322	0	152.18	-3.13	
323	0	152.18	-5.13	
324	0	152.18	-6.13	
325	0	152.18	-8.13	
326	0	152.18	-9.13	
327	0	152.18	-11.13	
328	0	152.18	-12.13	
329	0	153.05	-2.63	
330	0	153.05	-3.63	
331	0	153.05	-4.63	
332	0	153.05	-5.63	
333	0	153.05	-8.63	
334	0	153.05	-9.63	
335	0	153.05	-11.63	
336	0	153.91	-3.13	
337	0	153.91	-5.13	
338	0	153.91	-6.13	
339	0	153.91	-9.13	
340	0	153.91	-10.13	
341	0	153.91	-11.13	
342	0	153.91	-12.13	
343	0	154.78	-2.63	
344	6	154.78	-3.63	
345	0	154.78	-4.63	
346	0	154.78	-5.63	
347	0	154.78	-6.63	
348	0	154.78	-11.63	
349	0	155.65	-4.13	
350	0	155.65	-5.13	
351	0	155.65	-10.13	
352	0	155.65	-11.13	
353	0	156.51	-4.63	
354	0	156.51	-10.63	
355	0	157.38	-4.13	
356	0	157.38	-5.13	
357	0	159.11	-4.13	
358	0	159.98	-4.63	
359	0	180.49	-15.63	
360	0	180.49	-16.63	
361	0	180.49	-19.63	
362	0	180.49	-20.63	
363	0	181.36	-16.13	
364	0	181.36	-17.13	
365	0	181.36	-18.13	
366	0	181.36	-19.13	
367	0	181.36	-20.13	
368	0	181.36	-21.13	
369	0	182.23	-17.63	
370	0	182.23	-18.63	
371	0	182.23	-19.63	
372	0	173.83	-21.63	
373	0	174.7	-21.13	
374	0	174.7	-22.13	
375	0	176.43	-12.13	
376	0	176.43	-13.13	
377	0	176.43	-17.13	
378	0	176.43	-18.13	
379	0	177.3	-12.63	
380	0	177.3	-16.63	
381	0	177.3	-17.63	
382	0	177.3	-18.63	
383	0	178.16	-16.13	
384	0	178.16	-19.13	
385	0	179.03	-15.63	
386	0	179.03	-16.63	
387	0	179.03	-19.63	
388	0	179.9	-15.13	
389	0	179.9	-16.13	
390	0	179.9	-19.13	
391	0	179.9	-20.13	
392	0	175.57	-5.63	
393	0	176.43	-5.13	
394	0	176.43	-6.13	
395	0	176.43	-7.13	
396	0	177.3	-5.63	
397	0	177.3	-6.63	
398	0	177.3	-7.63	
399	0	178.16	-7.13	
400	0	178.16	-8.13	
401	0	179.03	-7.63	
402	0	179.03	-8.63	
403	0	179.03	-9.63	
404	0	179.03	-10.63	
405	0	179.9	-9.13	
406	0	179.9	-10.13	
407	0	179.9	-11.13	
408	0	124.47	37.87	
409	0	124.47	34.87	
410	0	124.47	33.87	
411	0	125.34	37.37	
412	0	125.34	36.37	
413	0	125.34	35.37	
414	0	125.34	34.37	
415	0	125.34	33.37	
416	0	126.2	35.87	
417	0	126.2	34.87	
418	0	126.2	33.87	
419	0	126.2	32.87	
420	0	127.07	34.37	
421	0	127.07	33.37	
422	0	127.93	38.87	
423	0	127.93	34.87	
424	0	127.93	33.87	
425	0	128.8	38.37	
426	0	128.8	37.37	
427	0	128.8	36.37	
428	0	128.8	35.37	
429	0	128.8	34.37	
430	0	129.67	37.87	
431	0	129.67	36.87	
432	0	129.67	35.87	
433	0	129.67	34.87	
434	0	130.53	37.37	
435	0	131.4	37.87	
436	0	123.6	39.37	
437	0	124.47	39.87	
438	0	124.47	38.87	
439	0	125.34	40.37	
440	0	125.34	39.37	
441	0	125.34	38.37	
442	0	126.2	37.87	
443	0	127.07	39.37	
444	0	127.93	39.87	
445	0	128.8	40.37	
446	0	129.67	41.87	
447	0	129.67	40.87	
448	0	130.53	42.37	
449	0	130.53	41.37	
450	0	101.95	10.87	
451	0	102.82	11.37	
452	0	102.82	10.37	
453	0	102.82	9.37	
454	0	97.62	9.37	
455	0	97.62	8.37	
456	0	97.62	7.37	
457	0	98.49	10.87	
458	0	98.49	9.87	
459	0	98.49	8.87	
460	0	98.49	7.87	
461	0	98.49	6.87	
462	0	99.36	12.37	
463	0	99.36	11.37	
464	0	99.36	10.37	
465	0	99.36	7.37	
466	0	99.36	6.37	
467	0	100.22	12.87	
468	0	100.22	11.87	
469	0	100.22	10.87	
470	0	100.22	9.87	
471	0	100.22	8.87	
472	0	100.22	6.87	
473	0	101.09	12.37	
474	0	101.09	8.37	
475	0	101.09	7.37	
476	0	101.95	12.87	
477	0	101.95	11.87	
478	0	101.95	7.87	
479	0	101.95	6.87	
480	0	101.95	5.87	
481	0	102.82	12.37	
482	0	91.56	11.87	
483	0	91.56	10.87	
484	0	92.43	13.37	
485	0	92.43	12.37	
486	0	92.43	11.37	
487	0	92.43	10.37	
488	0	92.43	9.37	
489	0	92.43	8.37	
490	0	93.29	13.87	
491	0	93.29	12.87	
492	0	93.29	11.87	
493	0	93.29	10.87	
494	0	93.29	8.87	
495	0	93.29	7.87	
496	0	93.29	6.87	
497	0	94.16	13.37	
498	0	94.16	12.37	
499	0	94.16	8.37	
500	0	94.16	7.37	
501	0	94.16	6.37	
502	7663	250.64	10.87	
503	7342	250.64	9.87	
504	32133	267.96	1.87	
505	23149	267.96	0.87	
506	4317	267.96	-0.13	
507	1609	267.96	-1.13	
508	245	268.83	1.37	
509	23	268.83	0.37	
510	0	268.83	-0.63	
511	2037	268.83	-1.63	
512	253	269.7	0.87	
513	0	269.7	-0.13	
514	103	269.7	-1.13	
515	210	270.56	0.37	
516	31	270.56	-0.63	
517	849	270.56	-1.63	
518	0	279.22	24.37	
519	0	279.22	23.37	
520	0	280.09	26.87	
521	0	280.09	25.87	
522	0	280.09	24.87	
523	0	280.09	23.87	
524	0	280.09	22.87	
525	0	280.95	27.37	
526	0	280.95	26.37	
527	0	280.95	25.37	
528	0	280.95	24.37	
529	0	280.95	23.37	
530	0	280.95	22.37	
531	0	281.82	27.87	
532	0	281.82	26.87	
533	0	281.82	22.87	
534	0	282.69	27.37	
535	0	282.69	26.37	
536	0	282.69	22.37	
537	0	283.55	26.87	
538	0	283.55	25.87	
539	0	283.55	24.87	
540	0	283.55	21.87	
541	0	284.42	25.37	
542	0	284.42	24.37	
543	0	284.42	21.37	
544	0	285.28	24.87	
545	0	285.28	23.87	
546	0	285.28	21.87	
547	0	285.28	20.87	
548	0	286.15	24.37	
549	0	286.15	23.37	
550	0	286.15	21.37	
551	0	286.15	20.37	
552	0	287.02	22.87	
553	0	287.02	21.87	
554	0	287.02	20.87	
555	0	287.88	22.37	
556	0	287.88	21.37	
557	0	288.75	21.87	
558	0	288.75	20.87	
559	0	289.61	21.37	
560	0	274.89	22.87	
561	0	274.89	21.87	
562	0	275.76	23.37	
563	0	275.76	22.37	
564	0	275.76	21.37	
565	0	276.62	22.87	
566	0	276.62	21.87	
567	0	276.62	20.87	
568	0	277.49	23.37	
569	0	277.49	21.37	
570	0	278.36	23.87	
571	0	278.36	22.87	
572	0	278.36	21.87	
573	0	278.36	20.87	
574	0	279.22	21.37	
575	0	280.09	21.87	
576	0	280.09	20.87	
577	0	280.95	21.37	
578	0	280.95	20.37	
579	0	281.82	21.87	
580	0	281.82	20.87	
581	0	281.82	19.87	
582	0	282.69	21.37	
583	0	282.69	20.37	
584	0	282.69	19.37	
585	0	283.55	20.87	
586	0	283.55	19.87	
587	0	284.42	20.37	
588	0	284.42	19.37	
589	0	285.28	19.87	
590	0	278.36	19.87	
591	0	278.36	18.87	
592	0	279.22	19.37	
593	0	280.09	19.87	
594	0	285.28	18.87	
595	0	285.28	17.87	
596	0	286.15	19.37	
597	0	286.15	18.37	
598	0	286.15	17.37	
599	0	287.02	19.87	
600	0	287.02	18.87	
601	0	287.02	17.87	
602	0	287.88	20.37	
603	0	287.88	19.37	
604	0	287.88	18.37	
605	0	287.88	17.37	
606	0	288.75	19.87	
607	0	288.75	17.87	
608	0	288.75	16.87	
609	0	289.61	18.37	
610	0	289.61	17.37	
611	0	290.48	20.87	
612	0	290.48	19.87	
613	0	290.48	17.87	
614	0	291.35	20.37	
615	0	291.35	19.37	
616	0	291.35	18.37	
617	0	292.21	18.87	
618	0	294.81	18.37	
619	0	295.68	18.87	
620	0	295.68	17.87	
621	0	296.54	18.37	
622	0	297.41	18.87	
623	0	297.41	17.87	
624	0	296.54	17.37	
625	0	297.41	16.87	
626	0	298.27	18.37	
627	0	298.27	17.37	
628	0	298.27	16.37	
629	0	299.14	13.87	
630	0	298.27	15.37	
631	0	299.14	15.87	
632	0	299.14	14.87	
633	0	300.01	13.37	
634	0	300.01	12.37	
635	0	300.87	12.87	
636	0	297.41	11.87	
637	0	298.27	12.37	
638	0	298.27	11.37	
639	0	297.41	9.87	
640	0	298.27	10.37	
641	0	299.14	11.87	
642	0	299.14	10.87	
643	0	299.14	9.87	
644	0	300.01	11.37	
645	0	298.27	13.37	
646	0	299.14	12.87	
647	41	276.62	7.87	
648	0	277.49	9.37	
649	0	277.49	8.37	
650	635	277.49	7.37	
651	0	278.36	9.87	
652	0	278.36	8.87	
653	1124	278.36	6.87	
654	0	279.22	9.37	
655	161	279.22	7.37	
656	0	280.09	9.87	
657	945	280.09	6.87	
658	0	280.95	9.37	
659	46	280.95	7.37	
660	0	281.82	9.87	
661	0	281.82	8.87	
662	28	281.82	6.87	
663	0	282.69	9.37	
664	0	282.69	8.37	
665	1125	272.29	5.37	
666	410	273.16	10.87	
667	819	273.16	5.87	
668	2589	273.16	4.87	
669	0	274.03	11.37	
670	44	274.03	10.37	
671	265	274.03	9.37	
672	0	274.89	9.87	
673	34	274.89	8.87	
674	0	275.76	9.37	
675	25	275.76	8.37	
676	0	276.62	10.87	
677	0	276.62	9.87	
678	0	276.62	8.87	
679	0	277.49	10.37	
680	0	272.29	13.37	
681	153	272.29	12.37	
682	0	273.16	12.87	
683	84	273.16	11.87	
684	0	276.62	14.87	
685	0	276.62	13.87	
686	0	276.62	12.87	
687	0	276.62	11.87	
688	0	277.49	15.37	
689	0	277.49	14.37	
690	0	277.49	13.37	
691	0	277.49	12.37	
692	0	278.36	13.87	
693	0	278.36	12.87	
694	0	278.36	11.87	
695	993	278.36	3.87	
696	0	279.22	13.37	
697	0	280.09	13.87	
698	0	280.09	12.87	
699	8	280.95	2.37	
700	0	280.95	1.37	
701	86	281.82	5.87	
702	57	281.82	4.87	
703	48	281.82	3.87	
704	0	281.82	2.87	
705	0	282.69	6.37	
706	0	282.69	5.37	
707	0	282.69	4.37	
708	0	283.55	9.87	
709	0	283.55	8.87	
710	0	284.42	11.37	
711	0	284.42	10.37	
712	0	285.28	11.87	
713	0	285.28	10.87	
714	0	286.15	12.37	
715	0	286.15	11.37	
716	0	287.02	11.87	
717	0	287.88	12.37	
718	0	288.75	12.87	
719	0	288.75	11.87	
720	0	271.43	15.87	
721	0	272.29	16.37	
722	0	272.29	15.37	
723	0	273.16	16.87	
724	0	273.16	15.87	
725	0	274.03	16.37	
726	0	274.89	16.87	
727	0	274.89	15.87	
728	0	275.76	17.37	
729	0	275.76	16.37	
730	0	275.76	15.37	
731	0	276.62	15.87	
732	0	277.49	16.37	
733	0	269.7	13.87	
734	632	269.7	12.87	
735	44	270.56	13.37	
736	85	271.43	12.87	
737	471	241.12	29.37	
738	2513	241.12	28.37	
739	124	241.98	29.87	
740	692	241.98	28.87	
741	4	242.85	32.37	
742	61	242.85	31.37	
743	0	243.71	31.87	
744	4	243.71	30.87	
745	259	243.71	29.87	
746	1548	243.71	24.87	
747	0	244.58	30.37	
748	82	244.58	29.37	
749	36	244.58	28.37	
750	113	244.58	27.37	
751	184	244.58	25.37	
752	2229	244.58	24.37	
753	4420	244.58	18.37	
754	0	245.45	28.87	
755	0	245.45	27.87	
756	195	245.45	26.87	
757	1188	245.45	18.87	
758	2735	245.45	17.87	
759	0	246.31	28.37	
760	0	246.31	27.37	
761	183	246.31	26.37	
762	0	247.18	28.87	
763	0	247.18	27.87	
764	0	247.18	26.87	
765	2	247.18	25.87	
766	148	247.18	24.87	
767	0	248.04	28.37	
768	0	248.04	27.37	
769	0	248.04	26.37	
770	0	248.04	25.37	
771	91	248.04	24.37	
772	0	248.91	27.87	
773	0	248.91	26.87	
774	0	248.91	25.87	
775	0	248.91	24.87	
776	82	248.91	23.87	
777	346	248.91	19.87	
778	1215	248.91	18.87	
779	0	249.78	27.37	
780	0	249.78	26.37	
781	0	249.78	25.37	
782	0	249.78	24.37	
783	9	249.78	23.37	
784	252	249.78	22.37	
785	575	249.78	19.37	
786	0	250.64	26.87	
787	0	250.64	25.87	
788	0	250.64	24.87	
789	7	250.64	23.87	
790	131	250.64	22.87	
791	0	251.51	25.37	
792	1	251.51	24.37	
793	0	252.37	24.87	
794	26	252.37	23.87	
795	0	253.24	24.37	
796	0	253.24	23.37	
797	11	253.24	22.37	
798	107	253.24	21.37	
799	0	254.11	22.87	
800	0	254.11	21.87	
801	29	254.11	20.87	
802	178	254.11	19.87	
803	0	254.97	21.37	
804	0	254.97	20.37	
805	83	254.97	19.37	
806	39	255.84	18.87	
807	21	256.71	18.37	
808	360	256.71	17.37	
809	21	257.57	17.87	
810	87	258.44	17.37	
811	106	259.3	16.87	
812	0	260.17	17.37	
813	201	260.17	16.37	
814	0	261.04	16.87	
815	365	261.04	15.87	
816	0	261.9	25.37	
817	0	261.9	24.37	
818	0	261.9	23.37	
819	0	261.9	22.37	
820	0	261.9	21.37	
821	0	261.9	16.37	
822	834	261.9	15.37	
823	0	262.77	25.87	
824	0	262.77	24.87	
825	0	262.77	23.87	
826	0	262.77	22.87	
827	0	262.77	21.87	
828	0	262.77	20.87	
829	0	262.77	19.87	
830	87	262.77	15.87	
831	0	263.63	20.37	
832	0	263.63	19.37	
833	0	263.63	18.37	
834	820	263.63	15.37	
835	0	264.5	18.87	
836	0	264.5	17.87	
837	239	264.5	15.87	
838	0	265.37	18.37	
839	0	265.37	17.37	
840	55	265.37	16.37	
841	0	266.23	18.87	
842	0	266.23	17.87	
843	104	266.23	15.87	
844	0	267.1	21.37	
845	0	267.1	18.37	
846	35	267.1	15.37	
847	422	267.1	14.37	
848	0	267.96	21.87	
849	0	267.96	20.87	
850	0	267.96	19.87	
851	0	267.96	18.87	
852	0	267.96	17.87	
853	0	267.96	14.87	
854	0	268.83	22.37	
855	0	268.83	20.37	
856	0	268.83	19.37	
857	0	268.83	18.37	
858	0	269.7	22.87	
859	0	269.7	21.87	
860	0	269.7	20.87	
861	0	269.7	19.87	
862	0	270.56	22.37	
863	0	270.56	21.37	
864	0	271.43	21.87	
865	0	272.29	21.37	
866	0	272.29	20.37	
867	0	272.29	19.37	
868	0	272.29	18.37	
869	0	273.16	21.87	
870	0	273.16	20.87	
871	0	273.16	19.87	
872	0	273.16	18.87	
873	264	267.96	13.87	
874	0	268.83	14.37	
875	342	268.83	13.37	
876	0	270.56	15.37	
877	294	278.36	-1.13	
878	310	278.36	-2.13	
879	338	278.36	-3.13	
880	75	279.22	0.37	
881	36	279.22	-0.63	
882	0	279.22	-1.63	
883	0	279.22	-2.63	
884	183	280.09	1.87	
885	2	280.09	0.87	
886	0	287.88	11.37	
887	0	288.75	10.87	
888	0	289.61	12.37	
889	0	289.61	11.37	
890	0	290.48	11.87	
891	0	291.35	11.37	
892	0	291.35	10.37	
893	0	292.21	11.87	
894	0	292.21	10.87	
895	0	293.08	12.37	
896	0	293.08	11.37	
897	0	293.08	10.37	
898	0	293.94	11.87	
899	0	293.94	10.87	
900	0	293.94	9.87	
901	0	294.81	12.37	
902	0	294.81	11.37	
903	0	294.81	10.37	
904	0	295.68	15.87	
905	0	295.68	11.87	
906	0	295.68	10.87	
907	0	295.68	9.87	
908	0	296.54	15.37	
909	0	296.54	11.37	
910	0	296.54	10.37	
911	0	297.41	10.87	
912	0	297.41	8.87	
913	0	298.27	9.37	
914	0	298.27	8.37	
915	0	299.14	8.87	
916	0	300.01	9.37	
917	0	300.01	8.37	
918	0	286.15	78.37	
919	0	286.15	77.37	
920	0	286.15	76.37	
921	0	287.02	78.87	
922	0	287.02	77.87	
923	0	287.02	76.87	
924	0	287.88	78.37	
925	0	287.88	77.37	
926	0	287.88	76.37	
927	0	288.75	78.87	
928	0	288.75	76.87	
929	0	288.75	75.87	
930	0	289.61	79.37	
931	0	289.61	78.37	
932	0	289.61	76.37	
933	0	290.48	78.87	
934	0	290.48	75.87	
935	0	291.35	80.37	
936	0	291.35	79.37	
937	0	291.35	76.37	
938	0	291.35	75.37	
939	0	292.21	80.87	
940	0	292.21	79.87	
941	0	292.21	78.87	
942	0	292.21	75.87	
943	0	293.08	80.37	
944	0	293.08	76.37	
945	0	293.08	75.37	
946	0	293.94	80.87	
947	0	293.94	75.87	
948	0	294.81	81.37	
949	0	294.81	76.37	
950	0	294.81	75.37	
951	0	295.68	80.87	
952	0	295.68	75.87	
953	0	296.54	81.37	
954	0	296.54	75.37	
955	0	297.41	81.87	
956	0	297.41	75.87	
957	0	298.27	82.37	
958	0	298.27	81.37	
959	0	298.27	75.37	
960	0	299.14	81.87	
961	0	299.14	75.87	
962	0	299.14	74.87	
963	0	300.01	82.37	
964	0	300.01	75.37	
965	0	300.87	81.87	
966	0	300.87	74.87	
967	0	300.87	73.87	
968	0	301.74	82.37	
969	0	301.74	74.37	
970	0	301.74	73.37	
971	0	302.6	82.87	
972	0	302.6	81.87	
973	0	302.6	73.87	
974	0	302.6	72.87	
975	0	303.47	82.37	
976	0	303.47	72.37	
977	0	303.47	71.37	
978	0	304.34	82.87	
979	0	304.34	81.87	
980	0	304.34	71.87	
981	0	304.34	70.87	
982	0	304.34	69.87	
983	0	304.85	82.37	
984	0	304.85	70.37	
985	0	304.85	69.37	
986	0	304.85	68.37	
987	0	304.85	67.37	
988	0	304.85	66.37	
989	0	228.99	-23.63	
990	0	228.99	-24.63	
991	0	229.86	-24.13	
992	0	229.86	-25.13	
993	0	231.59	-24.13	
994	0	235.05	-24.13	
995	0	235.05	-25.13	
996	0	235.92	-24.63	
997	0	204.74	-15.63	
998	0	204.74	-16.63	
999	0	204.74	-21.63	
1000	0	205.61	-15.13	
1001	0	205.61	-16.13	
1002	0	205.61	-17.13	
1003	0	205.61	-21.13	
1004	0	205.61	-22.13	
1005	0	206.48	-16.63	
1006	0	206.48	-22.63	
1007	0	207.34	-16.13	
1008	0	207.34	-17.13	
1009	0	207.34	-22.13	
1010	0	207.34	-23.13	
1011	0	208.21	-15.63	
1012	0	208.21	-16.63	
1013	0	208.21	-17.63	
1014	0	208.21	-22.63	
1015	0	209.07	-16.13	
1016	0	209.07	-17.13	
1017	0	209.07	-18.13	
1018	0	209.07	-22.13	
1019	0	209.94	-16.63	
1020	0	209.94	-17.63	
1021	0	209.94	-23.63	
1022	0	210.81	-15.13	
1023	0	210.81	-17.13	
1024	0	210.81	-18.13	
1025	0	210.81	-23.13	
1026	0	211.67	-14.63	
1027	0	211.67	-15.63	
1028	0	211.67	-17.63	
1029	0	211.67	-23.63	
1030	0	212.54	-15.13	
1031	0	212.54	-16.13	
1032	0	212.54	-24.13	
1033	0	213.4	-14.63	
1034	0	213.4	-15.63	
1035	0	213.4	-16.63	
1036	0	214.27	-14.13	
1037	0	214.27	-15.13	
1038	0	214.27	-16.13	
1039	0	214.27	-17.13	
1040	0	214.27	-18.13	
1041	0	214.27	-20.13	
1042	0	215.14	-14.63	
1043	0	215.14	-15.63	
1044	0	215.14	-16.63	
1045	0	215.14	-17.63	
1046	0	215.14	-19.63	
1047	0	215.14	-27.63	
1048	0	216	-15.13	
1049	0	216	-16.13	
1050	0	216	-17.13	
1051	0	216	-20.13	
1052	0	216	-21.13	
1053	0	216	-27.13	
1054	0	216	-28.13	
1055	0	216.87	-15.63	
1056	0	216.87	-16.63	
1057	0	216.87	-17.63	
1058	0	216.87	-20.63	
1059	0	216.87	-27.63	
1060	0	217.73	-14.13	
1061	0	217.73	-15.13	
1062	0	217.73	-16.13	
1063	0	217.73	-17.13	
1064	0	217.73	-18.13	
1065	0	217.73	-19.13	
1066	2	218.6	-8.63	
1067	0	218.6	-13.63	
1068	0	218.6	-14.63	
1069	0	218.6	-15.63	
1070	0	218.6	-16.63	
1071	0	218.6	-17.63	
1072	0	218.6	-18.63	
1073	0	218.6	-19.63	
1074	0	218.6	-21.63	
1075	7	219.47	-8.13	
1076	0	219.47	-9.13	
1077	0	219.47	-10.13	
1078	0	219.47	-14.13	
1079	0	219.47	-15.13	
1080	0	219.47	-16.13	
1081	0	219.47	-17.13	
1082	0	219.47	-18.13	
1083	0	219.47	-19.13	
1084	0	219.47	-20.13	
1085	0	219.47	-21.13	
1086	0	219.47	-22.13	
1087	1	220.33	-8.63	
1088	0	220.33	-9.63	
1089	0	220.33	-15.63	
1090	0	220.33	-18.63	
1091	0	220.33	-19.63	
1092	0	220.33	-20.63	
1093	0	220.33	-21.63	
1094	5	221.2	-9.13	
1095	0	221.2	-10.13	
1096	0	221.2	-11.13	
1097	0	221.2	-14.13	
1098	0	221.2	-15.13	
1099	0	221.2	-17.13	
1100	0	221.2	-18.13	
1101	0	221.2	-19.13	
1102	0	221.2	-20.13	
1103	0	221.2	-21.13	
1104	0	221.2	-22.13	
1105	0	222.06	-17.63	
1106	0	222.06	-20.63	
1107	0	222.93	-18.13	
1108	0	222.93	-21.13	
1109	0	222.93	-23.13	
1110	0	223.8	-18.63	
1111	0	223.8	-21.63	
1112	0	223.8	-22.63	
1113	0	224.66	-21.13	
1114	0	224.66	-22.13	
1115	0	224.66	-23.13	
1116	0	225.53	-22.63	
1117	0	225.53	-23.63	
1118	12702	199.55	5.37	
1119	4160	199.55	4.37	
1120	4982	200.41	3.87	
1121	2826	202.15	1.87	
1122	3637	203.01	2.37	
1123	2919	203.01	1.37	
1124	32	203.88	-5.13	
1125	1	203.88	-6.13	
1126	200	204.74	-3.63	
1127	79	204.74	-4.63	
1128	0	204.74	-5.63	
1129	48	205.61	-4.13	
1130	0	207.34	-10.13	
1131	0	208.21	-11.63	
1132	0	209.07	-10.13	
1133	0	209.94	-9.63	
1134	0	209.94	-10.63	
1135	1510	199.55	-0.63	
1136	3478	200.41	-0.13	
1137	15001	197.82	6.37	
1138	3696	197.82	5.37	
1139	0	188.29	-11.13	
1140	0	189.16	-10.63	
1141	0	189.16	-11.63	
1142	0	189.16	-13.63	
1143	0	189.16	-14.63	
1144	0	190.02	-14.13	
1145	0	190.89	-13.63	
1146	0	190.89	-14.63	
1147	0	191.75	-14.13	
1148	0	191.75	-15.13	
1149	0	186.56	-13.13	
1150	0	187.42	-13.63	
1151	0	188.29	-13.13	
1152	0	188.29	-14.13	
1153	0	193.49	-11.13	
1154	0	194.35	-10.63	
1155	0	194.35	-11.63	
1156	0	196.95	-13.13	
1157	0	196.95	-18.13	
1158	0	198.68	-10.13	
1159	0	198.68	-11.13	
1160	0	199.55	-10.63	
1161	0	199.55	-18.63	
1162	0	199.55	-21.63	
1163	0	200.41	-18.13	
1164	0	200.41	-19.13	
1165	0	200.41	-21.13	
1166	0	201.28	-8.63	
1167	0	201.28	-18.63	
1168	0	201.28	-19.63	
1169	0	202.15	-9.13	
1170	0	202.15	-20.13	
1171	0	202.15	-22.13	
1172	0	203.01	-19.63	
1173	0	203.01	-20.63	
1174	0	190.02	-19.13	
1175	0	183.09	-22.13	
1176	0	183.96	-15.63	
1177	0	183.96	-20.63	
1178	0	183.96	-22.63	
1179	0	184.82	-15.13	
1180	0	184.82	-16.13	
1181	0	184.82	-19.13	
1182	0	184.82	-20.13	
1183	0	184.82	-21.13	
1184	0	184.82	-22.13	
1185	0	185.69	-15.63	
1186	0	185.69	-17.63	
1187	0	185.69	-18.63	
1188	0	185.69	-19.63	
1189	0	185.69	-20.63	
1190	0	185.69	-21.63	
1191	0	186.56	-16.13	
1192	0	186.56	-19.13	
1193	0	187.42	-8.63	
1194	0	188.29	-9.13	
1195	0	189.16	-9.63	
1196	213	185.69	-3.63	
1197	43	185.69	-4.63	
1198	10	187.42	-4.63	
1199	111	188.29	-2.13	
1200	56	188.29	-3.13	
1201	2	188.29	-4.13	
1202	14	188.29	-5.13	
1203	22	189.16	-2.63	
1204	1	189.16	-3.63	
1205	0	189.16	-4.63	
1206	1367	183.09	0.87	
1207	580	183.09	-0.13	
1208	1612	183.96	0.37	
1209	43248	190.02	16.87	
1210	28405	190.89	17.37	
1211	17317	190.89	16.37	
1212	0	181.36	-14.13	
1213	0	182.23	-13.63	
1214	0	182.23	-14.63	
1215	0	183.09	-13.13	
1216	0	183.96	-12.63	
1217	0	183.96	-13.63	
1218	0	235.05	48.87	
1219	2	235.05	47.87	
1220	71	235.05	46.87	
1221	127	235.05	44.87	
1222	139	235.05	43.87	
1223	223	235.05	42.87	
1224	234	235.05	41.87	
1225	150	235.05	40.87	
1226	738	235.05	39.87	
1227	0	235.92	48.37	
1228	0	235.92	47.37	
1229	2	235.92	46.37	
1230	0	235.92	45.37	
1231	0	235.92	44.37	
1232	0	235.92	43.37	
1233	0	235.92	42.37	
1234	0	235.92	41.37	
1235	0	235.92	40.37	
1236	218	235.92	39.37	
1237	963	235.92	38.37	
1238	0	236.79	48.87	
1239	0	236.79	47.87	
1240	0	236.79	46.87	
1241	0	236.79	45.87	
1242	0	236.79	43.87	
1243	0	236.79	38.87	
1244	690	236.79	37.87	
1245	0	237.65	48.37	
1246	0	237.65	47.37	
1247	0	237.65	45.37	
1248	0	237.65	38.37	
1249	109	237.65	37.37	
1250	1221	237.65	36.37	
1251	0	238.52	37.87	
1252	0	238.52	36.87	
1253	126	238.52	35.87	
1254	1070	238.52	34.87	
1255	0	239.38	35.37	
1256	177	239.38	34.37	
1257	1099	239.38	33.37	
1258	0	240.25	34.87	
1259	0	240.25	33.87	
1260	491	240.25	32.87	
1261	0	241.12	34.37	
1262	0	241.12	33.37	
1263	239	241.12	32.37	
1264	0	241.98	33.87	
1265	3	241.98	32.87	
1266	0	242.85	33.37	
1267	0	261.9	27.37	
1268	0	262.77	28.87	
1269	0	262.77	27.87	
1270	0	262.77	26.87	
1271	0	263.63	28.37	
1272	0	264.5	29.87	
1273	0	264.5	28.87	
1274	0	265.37	29.37	
1275	0	266.23	29.87	
1276	0	267.1	30.37	
1277	0	267.1	29.37	
1278	0	267.96	29.87	
1279	0	267.96	28.87	
1280	0	268.83	30.37	
1281	0	268.83	29.37	
1282	0	268.83	28.37	
1283	0	269.7	30.87	
1284	0	269.7	29.87	
1285	0	269.7	28.87	
1286	0	270.56	30.37	
1287	0	270.56	29.37	
1288	0	270.56	28.37	
1289	0	271.43	30.87	
1290	0	271.43	29.87	
1291	0	271.43	28.87	
1292	0	272.29	31.37	
1293	0	272.29	30.37	
1294	0	273.16	30.87	
1295	0	273.16	29.87	
1296	0	274.03	30.37	
1297	0	274.03	29.37	
1298	0	274.89	30.87	
1299	0	274.89	29.87	
1300	0	275.76	30.37	
1301	0	275.76	29.37	
1302	0	276.62	29.87	
1303	0	276.62	28.87	
1304	0	276.62	27.87	
1305	0	276.62	24.87	
1306	0	277.49	29.37	
1307	0	277.49	28.37	
1308	0	277.49	27.37	
1309	0	277.49	26.37	
1310	0	277.49	24.37	
1311	0	278.36	31.87	
1312	0	278.36	30.87	
1313	0	278.36	29.87	
1314	0	278.36	28.87	
1315	0	278.36	26.87	
1316	0	278.36	25.87	
1317	0	278.36	24.87	
1318	0	279.22	32.37	
1319	0	279.22	31.37	
1320	0	279.22	30.37	
1321	0	279.22	29.37	
1322	0	279.22	28.37	
1323	0	279.22	27.37	
1324	0	279.22	25.37	
1325	0	280.09	32.87	
1326	0	280.09	31.87	
1327	0	280.09	27.87	
1328	0	280.95	33.37	
1329	0	280.95	32.37	
1330	0	281.82	39.87	
1331	0	281.82	38.87	
1332	0	281.82	37.87	
1333	0	281.82	34.87	
1334	0	281.82	33.87	
1335	0	282.69	41.37	
1336	0	282.69	40.37	
1337	0	282.69	39.37	
1338	0	282.69	38.37	
1339	0	282.69	37.37	
1340	0	282.69	36.37	
1341	0	282.69	35.37	
1342	0	282.69	34.37	
1343	0	283.55	40.87	
1344	0	283.55	39.87	
1345	0	283.55	38.87	
1346	0	283.55	37.87	
1347	0	283.55	36.87	
1348	0	283.55	35.87	
1349	0	283.55	34.87	
1350	0	284.42	41.37	
1351	0	284.42	40.37	
1352	0	284.42	39.37	
1353	0	284.42	38.37	
1354	0	284.42	37.37	
1355	0	284.42	36.37	
1356	0	284.42	35.37	
1357	0	285.28	40.87	
1358	0	285.28	39.87	
1359	0	285.28	38.87	
1360	0	285.28	37.87	
1361	0	286.15	42.37	
1362	0	286.15	41.37	
1363	0	286.15	40.37	
1364	0	286.15	39.37	
1365	0	287.02	41.87	
1366	0	287.02	40.87	
1367	0	287.88	41.37	
1368	0	287.88	40.37	
1369	0	288.75	42.87	
1370	0	288.75	41.87	
1371	0	288.75	40.87	
1372	0	289.61	44.37	
1373	0	289.61	43.37	
1374	0	289.61	42.37	
1375	0	289.61	41.37	
1376	0	290.48	44.87	
1377	0	290.48	43.87	
1378	0	290.48	41.87	
1379	0	290.48	40.87	
1380	0	291.35	44.37	
1381	0	291.35	43.37	
1382	0	292.21	44.87	
1383	0	292.21	43.87	
1384	0	293.08	45.37	
1385	0	293.08	44.37	
1386	0	271.43	17.87	
1387	0	272.29	17.37	
1388	0	280.95	18.37	
1389	0	280.95	17.37	
1390	0	281.82	18.87	
1391	0	281.82	17.87	
1392	0	281.82	16.87	
1393	0	282.69	18.37	
1394	0	282.69	17.37	
1395	0	282.69	16.37	
1396	0	283.55	18.87	
1397	0	283.55	17.87	
1398	0	283.55	16.87	
1399	0	284.42	17.37	
1400	0	300.87	8.87	
1401	0	300.87	7.87	
1402	0	300.87	6.87	
1403	0	301.74	7.37	
1404	0	301.74	6.37	
1405	0	302.6	6.87	
1406	0	302.6	5.87	
1407	0	303.47	6.37	
1408	0	303.47	5.37	
1409	0	304.34	5.87	
1410	0	304.85	6.37	
1411	0	180.49	52.37	
1412	0	180.49	51.37	
1413	0	181.36	51.87	
1414	4	181.36	50.87	
1415	0	182.23	52.37	
1416	2	182.23	51.37	
1417	2	183.09	51.87	
1418	0	183.96	52.37	
1419	14	183.96	51.37	
1420	0	184.82	52.87	
1421	1	184.82	51.87	
1422	0	185.69	52.37	
1423	16	185.69	51.37	
1424	0	186.56	60.87	
1425	0	186.56	59.87	
1426	3	186.56	51.87	
1427	0	187.42	63.37	
1428	0	187.42	60.37	
1429	0	187.42	52.37	
1430	0	188.29	63.87	
1431	0	188.29	62.87	
1432	0	188.29	60.87	
1433	0	188.29	59.87	
1434	0	188.29	52.87	
1435	13	188.29	51.87	
1436	0	189.16	64.37	
1437	0	189.16	63.37	
1438	0	189.16	57.37	
1439	0	189.16	56.37	
1440	0	189.16	53.37	
1441	5	189.16	52.37	
1442	0	190.02	63.87	
1443	0	190.02	62.87	
1444	0	190.02	57.87	
1445	0	190.02	56.87	
1446	0	190.02	55.87	
1447	1	190.02	52.87	
1448	0	190.89	66.37	
1449	0	190.89	65.37	
1450	0	190.89	63.37	
1451	0	190.89	62.37	
1452	0	190.89	56.37	
1453	0	190.89	53.37	
1454	25	190.89	52.37	
1455	0	191.75	65.87	
1456	0	191.75	64.87	
1457	0	191.75	63.87	
1458	0	191.75	62.87	
1459	0	191.75	59.87	
1460	0	191.75	53.87	
1461	2	191.75	52.87	
1462	0	192.62	68.37	
1463	0	192.62	66.37	
1464	0	192.62	65.37	
1465	0	192.62	64.37	
1466	0	192.62	60.37	
1467	0	192.62	59.37	
1468	0	192.62	54.37	
1469	1	192.62	53.37	
1470	0	193.49	68.87	
1471	0	193.49	67.87	
1472	0	193.49	66.87	
1473	0	193.49	65.87	
1474	0	193.49	64.87	
1475	0	193.49	63.87	
1476	0	193.49	61.87	
1477	0	193.49	60.87	
1478	0	193.49	59.87	
1479	0	193.49	54.87	
1480	0	193.49	53.87	
1481	23	193.49	52.87	
1482	0	194.35	69.37	
1483	0	194.35	68.37	
1484	0	194.35	67.37	
1485	0	194.35	66.37	
1486	0	194.35	65.37	
1487	0	194.35	64.37	
1488	0	194.35	63.37	
1489	0	194.35	62.37	
1490	0	194.35	61.37	
1491	0	194.35	60.37	
1492	0	194.35	59.37	
1493	0	194.35	54.37	
1494	5	194.35	53.37	
1495	0	195.22	68.87	
1496	0	195.22	67.87	
1497	0	195.22	66.87	
1498	0	195.22	64.87	
1499	0	195.22	63.87	
1500	0	195.22	62.87	
1501	0	195.22	61.87	
1502	0	195.22	60.87	
1503	0	195.22	59.87	
1504	0	195.22	54.87	
1505	2	195.22	53.87	
1506	0	196.08	70.37	
1507	0	196.08	69.37	
1508	0	196.08	67.37	
1509	0	196.08	66.37	
1510	0	196.08	64.37	
1511	0	196.08	63.37	
1512	0	196.08	62.37	
1513	0	196.08	61.37	
1514	0	196.08	60.37	
1515	0	196.08	59.37	
1516	0	196.08	55.37	
1517	0	196.08	54.37	
1518	0	196.95	69.87	
1519	0	196.95	68.87	
1520	0	196.95	66.87	
1521	0	196.95	65.87	
1522	0	196.95	64.87	
1523	0	196.95	63.87	
1524	0	196.95	62.87	
1525	0	196.95	60.87	
1526	0	196.95	59.87	
1527	0	196.95	58.87	
1528	0	196.95	55.87	
1529	0	196.95	54.87	
1530	7	196.95	53.87	
1531	0	197.82	70.37	
1532	0	197.82	67.37	
1533	0	197.82	66.37	
1534	0	197.82	64.37	
1535	0	197.82	63.37	
1536	0	197.82	60.37	
1537	0	197.82	59.37	
1538	0	197.82	58.37	
1539	0	197.82	56.37	
1540	0	197.82	55.37	
1541	0	197.82	54.37	
1542	0	198.68	70.87	
1543	0	198.68	69.87	
1544	0	198.68	66.87	
1545	0	198.68	65.87	
1546	0	198.68	64.87	
1547	0	198.68	63.87	
1548	0	198.68	58.87	
1549	0	198.68	57.87	
1550	0	198.68	55.87	
1551	0	198.68	54.87	
1552	0	199.55	71.37	
1553	0	199.55	70.37	
1554	0	199.55	67.37	
1555	0	199.55	66.37	
1556	0	199.55	64.37	
1557	0	199.55	63.37	
1558	0	199.55	59.37	
1559	0	199.55	58.37	
1560	0	199.55	56.37	
1561	0	199.55	55.37	
1562	1	199.55	54.37	
1563	0	200.41	70.87	
1564	0	200.41	69.87	
1565	0	200.41	66.87	
1566	0	200.41	58.87	
1567	0	200.41	57.87	
1568	0	200.41	56.87	
1569	0	200.41	55.87	
1570	0	200.41	54.87	
1571	0	201.28	71.37	
1572	0	201.28	70.37	
1573	0	201.28	66.37	
1574	0	201.28	59.37	
1575	0	201.28	58.37	
1576	0	201.28	57.37	
1577	0	201.28	56.37	
1578	0	201.28	55.37	
1579	2	201.28	54.37	
1580	0	202.15	70.87	
1581	0	202.15	58.87	
1582	0	202.15	57.87	
1583	0	202.15	56.87	
1584	0	202.15	55.87	
1585	0	203.01	71.37	
1586	0	203.01	59.37	
1587	0	203.01	58.37	
1588	0	203.01	57.37	
1589	0	203.01	56.37	
1590	0	203.01	55.37	
1591	0	203.88	71.87	
1592	0	203.88	70.87	
1593	0	203.88	58.87	
1594	0	203.88	57.87	
1595	0	203.88	56.87	
1596	0	203.88	55.87	
1597	0	204.74	71.37	
1598	0	204.74	70.37	
1599	0	204.74	58.37	
1600	0	204.74	57.37	
1601	0	204.74	56.37	
1602	3	204.74	55.37	
1603	0	205.61	70.87	
1604	0	205.61	59.87	
1605	0	205.61	58.87	
1606	0	205.61	57.87	
1607	0	205.61	56.87	
1608	0	205.61	55.87	
1609	0	206.48	71.37	
1610	0	206.48	70.37	
1611	0	206.48	60.37	
1612	0	206.48	59.37	
1613	0	206.48	58.37	
1614	0	206.48	57.37	
1615	0	206.48	56.37	
1616	0	207.34	70.87	
1617	0	207.34	60.87	
1618	0	207.34	59.87	
1619	0	207.34	58.87	
1620	0	207.34	57.87	
1621	0	207.34	56.87	
1622	0	208.21	71.37	
1623	0	208.21	70.37	
1624	0	208.21	61.37	
1625	0	208.21	60.37	
1626	0	208.21	59.37	
1627	0	208.21	58.37	
1628	0	208.21	57.37	
1629	0	209.07	70.87	
1630	0	209.07	69.87	
1631	0	209.07	61.87	
1632	0	209.07	60.87	
1633	0	209.07	59.87	
1634	0	209.07	58.87	
1635	0	209.94	70.37	
1636	0	209.94	61.37	
1637	0	209.94	60.37	
1638	0	209.94	59.37	
1639	0	210.81	70.87	
1640	0	210.81	69.87	
1641	0	210.81	61.87	
1642	0	210.81	60.87	
1643	0	210.81	59.87	
1644	0	210.81	58.87	
1645	0	211.67	70.37	
1646	0	211.67	61.37	
1647	0	211.67	60.37	
1648	0	211.67	59.37	
1649	0	212.54	70.87	
1650	0	212.54	69.87	
1651	0	212.54	60.87	
1652	0	212.54	59.87	
1653	0	213.4	70.37	
1654	0	213.4	61.37	
1655	0	213.4	60.37	
1656	0	213.4	59.37	
1657	0	214.27	70.87	
1658	0	214.27	69.87	
1659	0	214.27	60.87	
1660	0	214.27	59.87	
1661	0	214.27	58.87	
1662	0	215.14	70.37	
1663	0	215.14	60.37	
1664	0	215.14	59.37	
1665	0	216	69.87	
1666	0	216	59.87	
1667	0	216.87	70.37	
1668	0	216.87	60.37	
1669	0	216.87	59.37	
1670	0	217.73	69.87	
1671	0	217.73	59.87	
1672	0	218.6	70.37	
1673	0	218.6	69.37	
1674	0	218.6	60.37	
1675	0	218.6	59.37	
1676	0	219.47	69.87	
1677	0	219.47	59.87	
1678	0	220.33	60.37	
1679	0	220.33	59.37	
1680	0	221.2	59.87	
1681	0	221.2	58.87	
1682	0	222.06	59.37	
1683	0	222.06	58.37	
1684	0	222.93	58.87	
1685	0	222.93	57.87	
1686	0	223.8	59.37	
1687	0	223.8	58.37	
1688	0	223.8	57.37	
1689	0	223.8	56.37	
1690	0	224.66	59.87	
1691	0	224.66	58.87	
1692	0	224.66	57.87	
1693	0	224.66	56.87	
1694	0	224.66	55.87	
1695	0	225.53	58.37	
1696	0	225.53	57.37	
1697	0	225.53	56.37	
1698	0	225.53	55.37	
1699	0	226.39	58.87	
1700	0	226.39	57.87	
1701	0	226.39	56.87	
1702	0	226.39	55.87	
1703	0	226.39	54.87	
1704	0	227.26	58.37	
1705	0	227.26	57.37	
1706	0	227.26	56.37	
1707	0	227.26	55.37	
1708	0	227.26	54.37	
1709	0	228.13	56.87	
1710	0	228.13	55.87	
1711	0	228.13	54.87	
1712	0	228.99	56.37	
1713	0	228.99	55.37	
1714	0	228.99	54.37	
1715	0	229.86	55.87	
1716	0	229.86	54.87	
1717	1	172.1	53.37	
1718	17	172.1	52.37	
1719	0	172.97	52.87	
1720	24	172.97	51.87	
1721	0	173.83	53.37	
1722	2	173.83	52.37	
1723	0	174.7	52.87	
1724	1	175.57	52.37	
1725	0	176.43	52.87	
1726	2	176.43	51.87	
1727	0	177.3	52.37	
1728	16	177.3	51.37	
1729	0	178.16	51.87	
1730	0	179.03	52.37	
1731	0	179.03	51.37	
1732	0	179.9	51.87	
1733	6	179.9	50.87	
1734	0	279.22	-33.63	
1735	0	280.95	-33.63	
1736	0	283.55	-48.13	
1737	0	283.55	-49.13	
1738	0	283.55	-50.13	
1739	0	284.42	-43.63	
1740	0	284.42	-44.63	
1741	0	284.42	-45.63	
1742	0	284.42	-46.63	
1743	0	284.42	-47.63	
1744	0	284.42	-48.63	
1745	0	284.42	-49.63	
1746	0	284.42	-50.63	
1747	0	284.42	-51.63	
1748	0	284.42	-52.63	
1749	0	285.28	-38.13	
1750	0	285.28	-41.13	
1751	0	285.28	-42.13	
1752	0	285.28	-43.13	
1753	0	285.28	-44.13	
1754	0	285.28	-45.13	
1755	0	285.28	-52.13	
1756	0	285.28	-53.13	
1757	0	285.28	-54.13	
1758	0	286.15	-36.63	
1759	0	286.15	-37.63	
1760	0	286.15	-38.63	
1761	0	286.15	-39.63	
1762	0	286.15	-40.63	
1763	0	286.15	-41.63	
1764	0	286.15	-42.63	
1765	0	286.15	-52.63	
1766	0	286.15	-53.63	
1767	0	286.15	-54.63	
1768	0	287.02	-35.13	
1769	0	287.02	-36.13	
1770	0	287.02	-37.13	
1771	0	287.02	-38.13	
1772	0	287.02	-39.13	
1773	0	287.02	-40.13	
1774	0	287.02	-53.13	
1775	0	287.02	-54.13	
1776	0	287.02	-55.13	
1777	0	287.88	-28.63	
1778	0	287.88	-29.63	
1779	0	287.88	-30.63	
1780	0	287.88	-31.63	
1781	0	287.88	-32.63	
1782	0	287.88	-33.63	
1783	0	287.88	-34.63	
1784	0	287.88	-35.63	
1785	0	287.88	-36.63	
1786	0	287.88	-53.63	
1787	0	287.88	-54.63	
1788	1	288.75	-23.13	
1789	1	288.75	-24.13	
1790	0	288.75	-25.13	
1791	1	288.75	-26.13	
1792	0	288.75	-27.13	
1793	0	288.75	-28.13	
1794	0	288.75	-29.13	
1795	0	288.75	-30.13	
1796	0	288.75	-31.13	
1797	0	288.75	-32.13	
1798	0	288.75	-33.13	
1799	0	288.75	-53.13	
1800	0	288.75	-54.13	
1801	0	288.75	-55.13	
1802	0	289.61	-18.63	
1803	0	289.61	-19.63	
1804	0	289.61	-20.63	
1805	0	289.61	-21.63	
1806	0	289.61	-22.63	
1807	0	289.61	-23.63	
1808	0	289.61	-24.63	
1809	0	289.61	-25.63	
1810	0	289.61	-26.63	
1811	0	289.61	-52.63	
1812	0	289.61	-53.63	
1813	0	289.61	-55.63	
1814	0	290.48	-52.13	
1815	0	290.48	-53.13	
1816	0	290.48	-55.13	
1817	0	290.48	-56.13	
1818	0	291.35	-52.63	
1819	0	291.35	-55.63	
1820	0	291.35	-56.63	
1821	0	292.21	-56.13	
1822	0	293.08	-55.63	
1823	0	293.08	-56.63	
1824	0	293.94	-55.13	
1825	0	290.48	-51.13	
1826	0	291.35	-50.63	
1827	0	291.35	-51.63	
1828	0	292.21	-49.13	
1829	0	292.21	-50.13	
1830	0	292.21	-52.13	
1831	0	292.21	-53.13	
1832	0	292.21	-54.13	
1833	0	293.08	-48.63	
1834	0	293.08	-49.63	
1835	0	293.08	-53.63	
1836	0	293.08	-54.63	
1837	0	293.94	-45.13	
1838	0	293.94	-46.13	
1839	0	293.94	-47.13	
1840	0	293.94	-48.13	
1841	0	293.94	-54.13	
1842	0	294.81	-43.63	
1843	0	294.81	-44.63	
1844	0	294.81	-45.63	
1845	0	294.81	-46.63	
1846	0	294.81	-47.63	
1847	0	294.81	-54.63	
1848	0	294.81	-55.63	
1849	0	295.68	-42.13	
1850	0	295.68	-43.13	
1851	0	295.68	-54.13	
1852	0	295.68	-55.13	
1853	0	296.54	-41.63	
1854	0	296.54	-42.63	
1855	0	296.54	-54.63	
1856	0	297.41	-40.13	
1857	0	297.41	-41.13	
1858	0	298.27	-38.63	
1859	0	298.27	-39.63	
1860	0	298.27	-40.63	
1861	0	299.14	-39.13	
1862	0	300.01	-38.63	
1863	0	300.87	-38.13	
1864	0	300.87	-39.13	
1865	0	301.74	-38.63	
1866	0	302.6	-37.13	
1867	0	302.6	-38.13	
1868	0	303.47	-35.63	
1869	0	303.47	-36.63	
1870	0	303.47	-37.63	
1871	0	304.34	-36.13	
1872	0	304.34	-35.13	
1873	0	304.85	-34.63	
1874	0	304.85	-35.63	
1875	0	90.3	21.87	
1876	0	90.69	21.37	
1877	0	91.56	21.87	
1878	0	91.56	20.87	
1879	0	92.43	21.37	
1880	0	92.43	20.37	
1881	0	92.43	19.37	
1882	0	93.29	19.87	
1883	0	93.29	18.87	
1884	0	93.29	17.87	
1885	0	93.29	14.87	
1886	0	94.16	18.37	
1887	0	94.16	17.37	
1888	0	94.16	16.37	
1889	0	94.16	15.37	
1890	0	95.02	15.87	
1891	0	95.02	14.87	
1892	0	95.89	15.37	
1893	0	95.89	14.37	
1894	0	96.76	14.87	
1895	0	96.76	11.87	
1896	0	97.62	14.37	
1897	0	97.62	13.37	
1898	0	97.62	12.37	
1899	0	97.62	11.37	
1900	0	97.62	10.37	
1901	0	103.69	1.87	
1902	0	103.69	0.87	
1903	0	104.55	1.37	
1904	0	220.33	69.37	
1905	0	221.2	69.87	
1906	0	221.2	68.87	
1907	0	222.06	69.37	
1908	0	222.93	69.87	
1909	0	222.93	68.87	
1910	0	223.8	69.37	
1911	0	224.66	69.87	
1912	0	225.53	70.37	
1913	0	225.53	69.37	
1914	0	226.39	69.87	
1915	0	226.39	53.87	
1916	0	226.39	52.87	
1917	0	227.26	70.37	
1918	0	227.26	69.37	
1919	0	227.26	53.37	
1920	0	227.26	52.37	
1921	0	228.13	69.87	
1922	0	228.13	52.87	
1923	0	228.13	51.87	
1924	0	228.99	70.37	
1925	0	228.99	52.37	
1926	0	228.99	51.37	
1927	0	229.86	70.87	
1928	0	229.86	69.87	
1929	0	229.86	51.87	
1930	0	229.86	50.87	
1931	0	230.72	70.37	
1932	0	230.72	51.37	
1933	0	230.72	50.37	
1934	0	231.59	70.87	
1935	0	231.59	50.87	
1936	3	231.59	49.87	
1937	0	232.46	71.37	
1938	0	232.46	50.37	
1939	8	232.46	49.37	
1940	0	233.32	72.87	
1941	0	233.32	71.87	
1942	0	233.32	49.87	
1943	28	233.32	48.87	
1944	0	234.19	74.37	
1945	0	234.19	73.37	
1946	0	234.19	72.37	
1947	0	234.19	49.37	
1948	33	234.19	48.37	
1949	0	235.05	74.87	
1950	0	235.05	73.87	
1951	0	235.05	72.87	
1952	0	235.92	76.37	
1953	0	235.92	75.37	
1954	0	235.92	74.37	
1955	0	235.92	73.37	
1956	0	236.79	76.87	
1957	0	236.79	75.87	
1958	0	236.79	74.87	
1959	0	237.65	76.37	
1960	0	238.52	76.87	
1961	0	239.38	77.37	
1962	0	239.38	76.37	
1963	0	240.25	77.87	
1964	0	240.25	76.87	
1965	0	241.12	77.37	
1966	0	241.98	77.87	
1967	0	242.85	77.37	
1968	0	243.71	77.87	
1969	0	244.58	78.37	
1970	0	244.58	77.37	
1971	0	245.45	78.87	
1972	0	245.45	77.87	
1973	0	246.31	78.37	
1974	0	247.18	78.87	
1975	0	248.04	79.37	
1976	0	248.04	78.37	
1977	0	248.91	78.87	
1978	0	249.78	79.37	
1979	0	249.78	78.37	
1980	0	250.64	78.87	
1981	0	251.51	79.37	
1982	0	252.37	79.87	
1983	0	252.37	78.87	
1984	0	253.24	79.37	
1985	0	254.11	79.87	
1986	0	254.11	78.87	
1987	0	254.97	79.37	
1988	0	255.84	79.87	
1989	0	256.71	80.37	
1990	0	256.71	79.37	
1991	0	257.57	79.87	
1992	0	258.44	80.37	
1993	0	259.3	79.87	
1994	0	260.17	80.37	
1995	0	261.04	80.87	
1996	0	261.04	79.87	
1997	0	261.9	80.37	
1998	0	262.77	80.87	
1999	0	262.77	79.87	
2000	0	263.63	81.37	
2001	0	263.63	80.37	
2002	0	264.5	81.87	
2003	0	264.5	80.87	
2004	0	265.37	81.37	
2005	0	266.23	81.87	
2006	0	267.1	82.37	
2007	0	267.1	81.37	
2008	0	267.96	81.87	
2009	0	268.83	82.37	
2010	0	268.83	81.37	
2011	0	269.7	81.87	
2012	0	270.56	82.37	
2013	0	271.43	81.87	
2014	0	272.29	82.37	
2015	0	273.16	82.87	
2016	0	273.16	81.87	
2017	0	274.03	82.37	
2018	0	274.89	82.87	
2019	0	275.76	82.37	
2020	0	276.62	82.87	
2021	0	277.49	83.37	
2022	0	277.49	82.37	
2023	0	278.36	82.87	
2024	0	279.22	83.37	
2025	0	279.22	82.37	
2026	0	279.22	74.37	
2027	0	280.09	82.87	
2028	0	280.09	74.87	
2029	0	280.09	73.87	
2030	0	280.95	83.37	
2031	0	280.95	76.37	
2032	0	280.95	75.37	
2033	0	280.95	74.37	
2034	0	280.95	73.37	
2035	0	281.82	82.87	
2036	0	281.82	77.87	
2037	0	281.82	76.87	
2038	0	281.82	75.87	
2039	0	281.82	74.87	
2040	0	281.82	73.87	
2041	0	282.69	83.37	
2042	0	282.69	77.37	
2043	0	282.69	76.37	
2044	0	282.69	73.37	
2045	0	283.55	82.87	
2046	0	283.55	77.87	
2047	0	283.55	76.87	
2048	0	283.55	73.87	
2049	0	283.55	72.87	
2050	0	284.42	83.37	
2051	0	284.42	78.37	
2052	0	284.42	77.37	
2053	0	284.42	73.37	
2054	0	284.42	72.37	
2055	0	285.28	82.87	
2056	0	285.28	78.87	
2057	0	285.28	77.87	
2058	0	285.28	72.87	
2059	0	285.28	71.87	
2060	0	286.15	83.37	
2061	0	286.15	79.37	
2062	0	286.15	72.37	
2063	0	286.15	71.37	
2064	0	287.02	82.87	
2065	0	287.02	79.87	
2066	0	287.02	71.87	
2067	0	287.88	83.37	
2068	0	287.88	79.37	
2069	0	287.88	71.37	
2070	0	288.75	82.87	
2071	0	288.75	79.87	
2072	0	288.75	71.87	
2073	0	288.75	70.87	
2074	0	289.61	83.37	
2075	0	289.61	80.37	
2076	0	289.61	71.37	
2077	0	290.48	82.87	
2078	0	290.48	80.87	
2079	0	290.48	79.87	
2080	0	290.48	70.87	
2081	0	291.35	83.37	
2082	0	291.35	71.37	
2083	0	291.35	70.37	
2084	0	292.21	82.87	
2085	0	292.21	70.87	
2086	0	292.21	69.87	
2087	0	293.08	83.37	
2088	0	293.08	81.37	
2089	0	293.08	70.37	
2090	0	293.08	69.37	
2091	0	293.08	68.37	
2092	0	293.08	46.37	
2093	0	293.08	43.37	
2094	0	293.94	82.87	
2095	0	293.94	69.87	
2096	0	293.94	68.87	
2097	0	293.94	67.87	
2098	0	293.94	45.87	
2099	0	293.94	44.87	
2100	0	293.94	43.87	
2101	0	293.94	42.87	
2102	0	294.81	83.37	
2103	0	294.81	68.37	
2104	0	294.81	61.37	
2105	0	294.81	46.37	
2106	0	294.81	45.37	
2107	0	294.81	44.37	
2108	0	294.81	43.37	
2109	0	295.68	82.87	
2110	0	295.68	81.87	
2111	0	295.68	67.87	
2112	0	295.68	64.87	
2113	0	295.68	63.87	
2114	0	295.68	62.87	
2115	0	295.68	61.87	
2116	0	295.68	60.87	
2117	0	295.68	59.87	
2118	0	295.68	45.87	
2119	0	295.68	44.87	
2120	0	295.68	43.87	
2121	0	296.54	83.37	
2122	0	296.54	82.37	
2123	0	296.54	67.37	
2124	0	296.54	65.37	
2125	0	296.54	64.37	
2126	0	296.54	63.37	
2127	0	296.54	62.37	
2128	0	296.54	60.37	
2129	0	296.54	59.37	
2130	0	296.54	45.37	
2131	0	296.54	44.37	
2132	0	297.41	82.87	
2133	0	297.41	67.87	
2134	0	297.41	66.87	
2135	0	297.41	65.87	
2136	0	297.41	64.87	
2137	0	297.41	58.87	
2138	0	297.41	57.87	
2139	0	297.41	44.87	
2140	0	298.27	83.37	
2141	0	298.27	67.37	
2142	0	298.27	66.37	
2143	0	298.27	65.37	
2144	0	298.27	58.37	
2145	0	298.27	57.37	
2146	0	298.27	45.37	
2147	0	298.27	44.37	
2148	0	299.14	82.87	
2149	0	299.14	66.87	
2150	0	299.14	65.87	
2151	0	299.14	57.87	
2152	0	299.14	56.87	
2153	0	299.14	55.87	
2154	0	299.14	46.87	
2155	0	299.14	45.87	
2156	0	299.14	44.87	
2157	0	299.14	43.87	
2158	0	300.01	56.37	
2159	0	300.01	55.37	
2160	0	300.01	47.37	
2161	0	300.01	46.37	
2162	0	300.01	45.37	
2163	0	300.01	44.37	
2164	0	300.01	43.37	
2165	0	300.87	55.87	
2166	0	300.87	54.87	
2167	0	300.87	47.87	
2168	0	300.87	46.87	
2169	0	300.87	45.87	
2170	0	300.87	43.87	
2171	0	301.74	55.37	
2172	0	301.74	47.37	
2173	0	302.6	54.87	
2174	0	302.6	47.87	
2175	0	302.6	46.87	
2176	0	303.47	54.37	
2177	0	303.47	53.37	
2178	0	303.47	47.37	
2179	0	304.34	53.87	
2180	0	304.34	52.87	
2181	0	304.34	51.87	
2182	0	304.34	50.87	
2183	0	304.34	49.87	
2184	0	304.34	46.87	
2185	0	304.85	53.37	
2186	0	304.85	52.37	
2187	0	304.85	51.37	
2188	0	304.85	50.37	
2189	0	304.85	49.37	
2190	0	304.85	46.37	
2191	0	303.47	46.37	
2192	0	250.64	-27.13	
2193	0	254.11	-26.13	
2194	0	254.97	-26.63	
2195	0	279.22	-26.63	
2196	0	280.09	-26.13	
2197	0	290.48	12.87	
2198	0	291.35	12.37	
2199	0	299.14	16.87	
2200	0	298.27	14.37	
2201	0	292.21	17.87	
2202	0	293.08	18.37	
2203	0	293.08	17.37	
2204	0	293.94	18.87	
2205	0	293.94	17.87	
2206	0	294.81	17.37	
2207	0	279.22	14.37	
2208	0	280.09	14.87	
2209	0	278.36	14.87	
2210	10	144.39	13.37	
2211	1	145.25	13.87	
2212	1	145.25	12.87	
2213	109	144.39	20.37	
2214	10	144.39	14.37	
2215	235	145.25	20.87	
2216	190	145.25	19.87	
2217	153	145.25	18.87	
2218	11	145.25	17.87	
2219	17	145.25	16.87	
2220	9	145.25	15.87	
2221	9	145.25	14.87	
2222	156	146.12	18.37	
2223	29	146.12	17.37	
2224	4	146.12	16.37	
2225	3	146.12	15.37	
2226	0	280.09	15.87	
2227	0	280.95	16.37	
2228	0	280.95	15.37	
2229	0	281.82	15.87	
2230	0	123.6	-9.63	
2231	0	124.47	-9.13	
2232	10	169.5	-1.13	
2233	96	172.1	3.37	
2234	8	172.1	2.37	
2235	3	172.1	1.37	
2236	3727	172.97	3.87	
2237	2154	172.97	2.87	
2238	0	172.97	1.87	
2239	12	172.97	0.87	
2240	11	172.97	-0.13	
2241	4137	173.83	2.37	
2242	1445	173.83	1.37	
2243	261	173.83	0.37	
2244	0	173.83	-0.63	
2245	302	174.7	-0.13	
2246	0	174.7	-1.13	
2247	2	174.7	-2.13	
2248	69	175.57	-0.63	
2249	0	175.57	-1.63	
2250	2	175.57	-2.63	
2251	15	176.43	-1.13	
2252	0	176.43	-2.13	
2253	0	176.43	-3.13	
2254	4	177.3	-2.63	
2255	0	180.49	71.37	
2256	0	180.49	70.37	
2257	0	180.49	69.37	
2258	0	180.49	68.37	
2259	0	180.49	67.37	
2260	0	180.49	65.37	
2261	0	181.36	71.87	
2262	0	181.36	70.87	
2263	0	181.36	68.87	
2264	0	181.36	67.87	
2265	0	181.36	66.87	
2266	0	181.36	65.87	
2267	0	181.36	64.87	
2268	0	182.23	71.37	
2269	0	182.23	70.37	
2270	0	182.23	68.37	
2271	0	182.23	67.37	
2272	0	182.23	65.37	
2273	0	183.09	71.87	
2274	0	183.09	70.87	
2275	0	183.09	68.87	
2276	0	183.09	67.87	
2277	0	183.09	65.87	
2278	0	183.09	64.87	
2279	0	183.96	68.37	
2280	0	183.96	67.37	
2281	0	183.96	65.37	
2282	0	183.96	64.37	
2283	0	184.82	67.87	
2284	0	184.82	64.87	
2285	0	185.69	67.37	
2286	0	185.69	64.37	
2287	0	186.56	67.87	
2288	0	186.56	66.87	
2289	0	186.56	63.87	
2290	0	187.42	67.37	
2291	0	187.42	65.37	
2292	0	187.42	64.37	
2293	0	188.29	66.87	
2294	0	188.29	65.87	
2295	0	188.29	64.87	
2296	0	189.16	67.37	
2297	0	189.16	66.37	
2298	0	189.16	65.37	
2299	0	190.02	66.87	
2300	0	190.02	65.87	
2301	0	90.3	81.87	
2302	0	90.3	80.87	
2303	0	90.3	79.87	
2304	0	90.3	78.87	
2305	0	90.3	77.87	
2306	0	90.3	76.87	
2307	0	90.3	75.87	
2308	0	90.69	81.37	
2309	0	90.69	80.37	
2310	0	90.69	79.37	
2311	0	90.69	77.37	
2312	0	90.69	76.37	
2313	0	91.56	81.87	
2314	0	91.56	80.87	
2315	0	91.56	79.87	
2316	0	91.56	78.87	
2317	0	91.56	77.87	
2318	0	91.56	76.87	
2319	0	91.56	75.87	
2320	0	92.43	81.37	
2321	0	92.43	80.37	
2322	0	92.43	79.37	
2323	0	92.43	78.37	
2324	0	92.43	77.37	
2325	0	92.43	76.37	
2326	0	93.29	80.87	
2327	0	93.29	78.87	
2328	0	93.29	77.87	
2329	0	93.29	76.87	
2330	0	93.29	75.87	
2331	0	94.16	81.37	
2332	0	94.16	79.37	
2333	0	94.16	78.37	
2334	0	94.16	77.37	
2335	0	94.16	76.37	
2336	0	95.02	81.87	
2337	0	95.02	80.87	
2338	0	95.02	78.87	
2339	0	95.02	77.87	
2340	0	95.02	76.87	
2341	0	95.89	81.37	
2342	0	95.89	79.37	
2343	0	95.89	78.37	
2344	0	95.89	77.37	
2345	0	96.76	81.87	
2346	0	96.76	80.87	
2347	0	96.76	78.87	
2348	0	96.76	77.87	
2349	0	96.76	76.87	
2350	0	97.62	81.37	
2351	0	97.62	80.37	
2352	0	97.62	78.37	
2353	0	97.62	77.37	
2354	0	98.49	80.87	
2355	0	98.49	79.87	
2356	0	98.49	77.87	
2357	0	99.36	80.37	
2358	0	99.36	78.37	
2359	0	99.36	77.37	
2360	0	100.22	79.87	
2361	0	101.09	80.37	
2362	0	101.09	79.37	
2363	0	101.95	79.87	
2364	0	102.82	79.37	
2365	0	103.69	79.87	
2366	0	103.69	78.87	
2367	0	104.55	79.37	
2368	0	105.42	78.87	
2369	0	106.28	79.37	
2370	0	106.28	78.37	
2371	0	107.15	78.87	
2372	0	107.15	77.87	
2373	0	108.02	78.37	
2374	0	108.02	77.37	
2375	0	108.88	77.87	
2376	0	108.88	76.87	
2377	0	109.75	77.37	
2378	0	109.75	76.37	
2379	0	110.61	76.87	
2380	0	111.48	77.37	
2381	0	111.48	76.37	
2382	0	112.35	76.87	
2383	0	112.35	74.87	
2384	0	112.35	73.87	
2385	0	113.21	76.37	
2386	0	113.21	75.37	
2387	0	113.21	74.37	
2388	0	113.21	73.37	
2389	0	114.08	76.87	
2390	0	114.08	75.87	
2391	0	114.08	74.87	
2392	0	114.08	73.87	
2393	0	114.94	76.37	
2394	0	114.94	75.37	
2395	0	114.94	74.37	
2396	0	114.94	73.37	
2397	0	115.81	74.87	
2398	0	115.81	73.87	
2399	0	116.68	74.37	
2400	0	116.68	73.37	
2401	0	117.54	73.87	
2402	0	118.41	73.37	
2403	0	119.27	73.87	
2404	0	119.27	72.87	
2405	0	120.14	73.37	
2406	0	121.01	72.87	
2407	0	121.87	73.37	
2408	0	122.74	73.87	
2409	0	122.74	72.87	
2410	0	123.6	74.37	
2411	0	123.6	73.37	
2412	0	124.47	73.87	
2413	0	125.34	74.37	
2414	0	125.34	73.37	
2415	0	126.2	73.87	
2416	0	127.07	73.37	
2417	0	127.93	73.87	
2418	0	128.8	73.37	
2419	0	129.67	72.87	
2420	0	129.67	71.87	
2421	0	129.67	70.87	
2422	0	130.53	73.37	
2423	0	130.53	72.37	
2424	0	130.53	71.37	
2425	0	131.4	71.87	
2426	0	131.4	70.87	
2427	0	131.4	42.87	
2428	0	131.4	41.87	
2429	0	132.26	72.37	
2430	0	132.26	71.37	
2431	0	132.26	70.37	
2432	0	132.26	69.37	
2433	0	132.26	68.37	
2434	0	132.26	42.37	
2435	0	133.13	71.87	
2436	0	133.13	70.87	
2437	0	133.13	69.87	
2438	0	133.13	68.87	
2439	0	133.13	42.87	
2440	0	134	72.37	
2441	0	134	71.37	
2442	0	134	69.37	
2443	0	134	68.37	
2444	0	134	43.37	
2445	0	134	42.37	
2446	0	134.86	75.87	
2447	0	134.86	74.87	
2448	0	134.86	73.87	
2449	0	134.86	71.87	
2450	0	134.86	70.87	
2451	0	134.86	69.87	
2452	0	134.86	68.87	
2453	0	134.86	67.87	
2454	0	134.86	43.87	
2455	0	134.86	42.87	
2456	0	135.73	76.37	
2457	0	135.73	75.37	
2458	0	135.73	74.37	
2459	0	135.73	73.37	
2460	0	135.73	71.37	
2461	0	135.73	70.37	
2462	0	135.73	44.37	
2463	0	135.73	43.37	
2464	0	136.59	75.87	
2465	0	136.59	74.87	
2466	0	136.59	73.87	
2467	0	136.59	71.87	
2468	0	136.59	70.87	
2469	0	136.59	44.87	
2470	0	137.46	76.37	
2471	0	137.46	75.37	
2472	0	137.46	74.37	
2473	0	137.46	72.37	
2474	0	137.46	71.37	
2475	0	137.46	56.37	
2476	0	137.46	55.37	
2477	0	137.46	46.37	
2478	0	137.46	45.37	
2479	0	138.33	76.87	
2480	0	138.33	75.87	
2481	0	138.33	74.87	
2482	0	138.33	72.87	
2483	0	138.33	71.87	
2484	0	138.33	56.87	
2485	0	138.33	55.87	
2486	0	138.33	54.87	
2487	0	138.33	46.87	
2488	0	138.33	45.87	
2489	0	139.19	76.37	
2490	0	139.19	74.37	
2491	0	139.19	73.37	
2492	0	139.19	72.37	
2493	0	139.19	57.37	
2494	0	139.19	56.37	
2495	0	139.19	55.37	
2496	0	139.19	54.37	
2497	0	139.19	48.37	
2498	0	139.19	47.37	
2499	0	140.06	76.87	
2500	0	140.06	75.87	
2501	0	140.06	73.87	
2502	0	140.06	72.87	
2503	0	140.06	71.87	
2504	0	140.06	57.87	
2505	0	140.06	56.87	
2506	0	140.06	54.87	
2507	0	140.06	53.87	
2508	0	140.06	50.87	
2509	0	140.06	49.87	
2510	0	140.06	48.87	
2511	0	140.06	47.87	
2512	0	140.92	76.37	
2513	0	140.92	74.37	
2514	0	140.92	58.37	
2515	0	140.92	57.37	
2516	0	140.92	54.37	
2517	0	140.92	53.37	
2518	0	140.92	52.37	
2519	0	140.92	51.37	
2520	0	140.92	50.37	
2521	0	140.92	49.37	
2522	0	140.92	48.37	
2523	0	140.92	46.37	
2524	0	141.79	75.87	
2525	0	141.79	74.87	
2526	0	141.79	73.87	
2527	0	141.79	58.87	
2528	0	141.79	57.87	
2529	1	141.79	54.87	
2530	0	141.79	53.87	
2531	0	141.79	52.87	
2532	0	141.79	51.87	
2533	0	141.79	50.87	
2534	0	141.79	49.87	
2535	0	141.79	48.87	
2536	0	141.79	47.87	
2537	0	141.79	46.87	
2538	0	141.79	45.87	
2539	0	142.66	76.37	
2540	0	142.66	75.37	
2541	0	142.66	74.37	
2542	0	142.66	73.37	
2543	0	142.66	59.37	
2544	0	142.66	56.37	
2545	0	142.66	54.37	
2546	0	142.66	53.37	
2547	0	142.66	52.37	
2548	0	142.66	51.37	
2549	0	142.66	49.37	
2550	0	142.66	48.37	
2551	0	142.66	47.37	
2552	0	142.66	46.37	
2553	0	143.52	75.87	
2554	0	143.52	74.87	
2555	0	143.52	73.87	
2556	0	143.52	72.87	
2557	0	143.52	59.87	
2558	0	143.52	58.87	
2559	0	143.52	56.87	
2560	2	143.52	55.87	
2561	0	143.52	53.87	
2562	1	143.52	52.87	
2563	0	143.52	51.87	
2564	0	143.52	50.87	
2565	0	143.52	49.87	
2566	0	143.52	48.87	
2567	2	143.52	46.87	
2568	0	143.52	45.87	
2569	0	144.39	76.37	
2570	0	144.39	75.37	
2571	0	144.39	74.37	
2572	0	144.39	73.37	
2573	0	144.39	72.37	
2574	0	144.39	60.37	
2575	0	144.39	59.37	
2576	0	144.39	50.37	
2577	0	144.39	49.37	
2578	1	144.39	48.37	
2579	25	144.39	46.37	
2580	0	145.25	75.87	
2581	0	145.25	74.87	
2582	0	145.25	72.87	
2583	0	145.25	59.87	
2584	0	145.25	58.87	
2585	5	145.25	48.87	
2586	0	146.12	75.37	
2587	0	146.12	72.37	
2588	0	146.12	59.37	
2589	0	146.99	75.87	
2590	0	146.99	74.87	
2591	0	146.99	72.87	
2592	0	146.99	71.87	
2593	0	146.99	58.87	
2594	0	147.85	76.37	
2595	0	147.85	75.37	
2596	0	147.85	74.37	
2597	0	147.85	72.37	
2598	0	147.85	59.37	
2599	0	148.72	76.87	
2600	0	148.72	75.87	
2601	0	148.72	74.87	
2602	0	148.72	72.87	
2603	0	148.72	71.87	
2604	0	148.72	58.87	
2605	340	148.72	45.87	
2606	0	149.58	77.37	
2607	0	149.58	76.37	
2608	0	149.58	75.37	
2609	0	149.58	74.37	
2610	0	149.58	72.37	
2611	0	149.58	59.37	
2612	0	149.58	58.37	
2613	1	149.58	46.37	
2614	3775	149.58	45.37	
2615	0	150.45	76.87	
2616	0	150.45	75.87	
2617	0	150.45	74.87	
2618	0	150.45	71.87	
2619	0	150.45	58.87	
2620	2	150.45	46.87	
2621	1597	150.45	45.87	
2622	0	151.32	76.37	
2623	0	151.32	75.37	
2624	0	151.32	74.37	
2625	0	151.32	71.37	
2626	0	151.32	59.37	
2627	0	151.32	58.37	
2628	0	151.32	47.37	
2629	1319	151.32	46.37	
2630	0	152.18	76.87	
2631	0	152.18	75.87	
2632	0	152.18	71.87	
2633	0	152.18	70.87	
2634	0	152.18	58.87	
2635	0	152.18	47.87	
2636	834	152.18	46.87	
2637	0	153.05	76.37	
2638	0	153.05	75.37	
2639	0	153.05	71.37	
2640	0	153.05	70.37	
2641	0	153.05	59.37	
2642	0	153.05	58.37	
2643	0	153.05	49.37	
2644	0	153.05	48.37	
2645	514	153.05	47.37	
2646	0	153.91	75.87	
2647	0	153.91	70.87	
2648	0	153.91	59.87	
2649	0	153.91	58.87	
2650	0	153.91	49.87	
2651	0	153.91	48.87	
2652	350	153.91	47.87	
2653	0	154.78	71.37	
2654	0	154.78	60.37	
2655	0	154.78	59.37	
2656	0	154.78	55.37	
2657	0	154.78	54.37	
2658	0	154.78	51.37	
2659	0	154.78	50.37	
2660	31	154.78	49.37	
2661	505	154.78	48.37	
2662	0	155.65	76.87	
2663	0	155.65	70.87	
2664	0	155.65	60.87	
2665	0	155.65	59.87	
2666	0	155.65	58.87	
2667	0	155.65	56.87	
2668	0	155.65	55.87	
2669	0	155.65	54.87	
2670	0	155.65	53.87	
2671	0	155.65	52.87	
2672	0	155.65	51.87	
2673	0	155.65	50.87	
2674	107	155.65	49.87	
2675	0	156.51	77.37	
2676	0	156.51	71.37	
2677	0	156.51	70.37	
2678	0	156.51	61.37	
2679	0	156.51	60.37	
2680	0	156.51	58.37	
2681	0	156.51	57.37	
2682	0	156.51	56.37	
2683	0	156.51	53.37	
2684	0	156.51	52.37	
2685	0	156.51	51.37	
2686	208	156.51	50.37	
2687	0	157.38	76.87	
2688	0	157.38	70.87	
2689	0	157.38	61.87	
2690	0	157.38	60.87	
2691	0	157.38	57.87	
2692	0	157.38	56.87	
2693	0	157.38	51.87	
2694	172	157.38	50.87	
2695	0	158.24	77.37	
2696	0	158.24	76.37	
2697	0	158.24	71.37	
2698	0	158.24	70.37	
2699	0	158.24	61.37	
2700	0	158.24	58.37	
2701	0	158.24	57.37	
2702	0	158.24	52.37	
2703	217	158.24	51.37	
2704	0	159.11	76.87	
2705	0	159.11	70.87	
2706	0	159.11	61.87	
2707	0	159.11	60.87	
2708	0	159.11	58.87	
2709	0	159.11	57.87	
2710	0	159.11	52.87	
2711	82	159.11	51.87	
2712	0	159.98	71.37	
2713	0	159.98	70.37	
2714	0	159.98	69.37	
2715	0	159.98	61.37	
2716	0	159.98	60.37	
2717	0	159.98	59.37	
2718	0	159.98	53.37	
2719	42	159.98	52.37	
2720	0	160.84	70.87	
2721	0	160.84	69.87	
2722	0	160.84	60.87	
2723	0	160.84	59.87	
2724	1	160.84	53.87	
2725	61	160.84	52.87	
2726	0	161.71	71.37	
2727	0	161.71	70.37	
2728	0	161.71	69.37	
2729	0	161.71	60.37	
2730	4	161.71	54.37	
2731	0	162.57	70.87	
2732	0	162.57	69.87	
2733	0	162.57	55.87	
2734	5	162.57	54.87	
2735	0	163.44	70.37	
2736	0	163.44	69.37	
2737	0	163.44	58.37	
2738	0	163.44	57.37	
2739	0	163.44	56.37	
2740	2	163.44	55.37	
2741	0	164.31	69.87	
2742	0	164.31	58.87	
2743	0	165.17	69.37	
2744	0	165.17	59.37	
2745	0	165.17	58.37	
2746	2	165.17	55.37	
2747	0	166.04	69.87	
2748	0	166.04	59.87	
2749	0	166.04	58.87	
2750	0	166.04	55.87	
2751	7	166.04	54.87	
2752	0	166.91	70.37	
2753	0	166.91	69.37	
2754	0	166.91	60.37	
2755	0	166.91	59.37	
2756	0	166.91	55.37	
2757	1	166.91	54.37	
2758	0	167.77	69.87	
2759	0	167.77	60.87	
2760	0	167.77	59.87	
2761	0	167.77	54.87	
2762	8	167.77	53.87	
2763	0	168.64	70.37	
2764	0	168.64	60.37	
2765	0	168.64	54.37	
2766	0	169.5	69.87	
2767	0	169.5	59.87	
2768	0	170.37	70.37	
2769	0	170.37	60.37	
2770	0	170.37	59.37	
2771	0	171.24	69.87	
2772	0	171.24	60.87	
2773	0	171.24	59.87	
2774	0	172.1	70.37	
2775	0	172.1	61.37	
2776	0	172.1	60.37	
2777	0	172.97	69.87	
2778	0	172.97	61.87	
2779	0	172.97	60.87	
2780	0	173.83	70.37	
2781	0	173.83	69.37	
2782	0	173.83	62.37	
2783	0	173.83	61.37	
2784	0	174.7	69.87	
2785	0	174.7	61.87	
2786	0	175.57	70.37	
2787	0	175.57	69.37	
2788	0	175.57	62.37	
2789	0	175.57	61.37	
2790	0	176.43	69.87	
2791	0	176.43	62.87	
2792	0	176.43	61.87	
2793	0	177.3	69.37	
2794	0	177.3	62.37	
2795	0	178.16	71.87	
2796	0	178.16	70.87	
2797	0	178.16	69.87	
2798	0	178.16	64.87	
2799	0	178.16	63.87	
2800	0	178.16	62.87	
2801	0	178.16	61.87	
2802	0	179.03	71.37	
2803	0	179.03	70.37	
2804	0	179.03	69.37	
2805	0	179.03	64.37	
2806	0	179.03	63.37	
2807	0	179.03	62.37	
2808	0	179.9	71.87	
2809	0	179.9	70.87	
2810	0	179.9	69.87	
2811	0	179.9	68.87	
2812	0	179.9	64.87	
2813	0	179.9	62.87	
2814	0	179.9	61.87	
2815	57	160.84	9.87	
2816	201	161.71	11.37	
2817	173	162.57	11.87	
2818	26	162.57	10.87	
2819	546	165.17	12.37	
2820	153	165.17	11.37	
2821	33	165.17	10.37	
2822	20	165.17	9.37	
2823	17	165.17	8.37	
2824	43	166.04	11.87	
2825	0	166.04	10.87	
2826	0	166.04	9.87	
2827	0	166.04	8.87	
2828	25	166.91	11.37	
2829	0	166.91	10.37	
2830	0	166.91	9.37	
2831	1	166.91	8.37	
2832	209	167.77	11.87	
2833	1	167.77	10.87	
2834	0	167.77	9.87	
2835	0	167.77	8.87	
2836	1	167.77	7.87	
2837	4	167.77	5.87	
2838	1692	168.64	15.37	
2839	867	168.64	14.37	
2840	1	168.64	10.37	
2841	0	168.64	9.37	
2842	0	168.64	8.37	
2843	1	168.64	7.37	
2844	1	168.64	6.37	
2845	28	168.64	5.37	
2846	506	168.64	4.37	
2847	294	169.5	14.87	
2848	245	169.5	11.87	
2849	19	169.5	10.87	
2850	0	169.5	9.87	
2851	0	169.5	8.87	
2852	0	169.5	7.87	
2853	0	169.5	6.87	
2854	3	169.5	5.87	
2855	414	169.5	4.87	
2856	398	170.37	12.37	
2857	1	170.37	11.37	
2858	0	170.37	10.37	
2859	0	170.37	9.37	
2860	0	170.37	8.37	
2861	0	170.37	7.37	
2862	11	171.24	10.87	
2863	0	171.24	9.87	
2864	0	171.24	8.87	
2865	3	171.24	7.87	
2866	0	171.24	6.87	
2867	7	171.24	5.87	
2868	92	172.1	7.37	
2869	537	172.1	6.37	
2870	896	172.1	5.37	
2871	0	180.49	-31.63	
2872	0	181.36	-29.13	
2873	0	181.36	-30.13	
2874	0	181.36	-31.13	
2875	0	182.23	-28.63	
2876	0	182.23	-29.63	
2877	0	182.23	-43.63	
2878	0	183.09	-43.13	
2879	0	183.09	-44.13	
2880	0	183.96	-43.63	
2881	0	183.96	-44.63	
2882	0	184.82	-44.13	
2883	0	165.17	-50.63	
2884	0	166.04	-45.13	
2885	0	166.04	-46.13	
2886	0	166.04	-48.13	
2887	0	166.04	-50.13	
2888	0	166.04	-51.13	
2889	0	166.91	-44.63	
2890	0	166.91	-45.63	
2891	0	166.91	-46.63	
2892	0	166.91	-47.63	
2893	0	166.91	-48.63	
2894	0	166.91	-50.63	
2895	0	167.77	-44.13	
2896	0	167.77	-45.13	
2897	0	167.77	-46.13	
2898	0	167.77	-47.13	
2899	0	167.77	-48.13	
2900	0	168.64	-43.63	
2901	0	168.64	-44.63	
2902	0	168.64	-46.63	
2903	0	168.64	-47.63	
2904	0	168.64	-52.63	
2905	0	169.5	-43.13	
2906	0	169.5	-44.13	
2907	0	169.5	-46.13	
2908	0	169.5	-47.13	
2909	0	169.5	-52.13	
2910	0	169.5	-53.13	
2911	0	170.37	-42.63	
2912	0	170.37	-43.63	
2913	0	170.37	-45.63	
2914	0	170.37	-46.63	
2915	0	171.24	-34.13	
2916	0	171.24	-41.13	
2917	0	171.24	-42.13	
2918	0	171.24	-43.13	
2919	0	171.24	-44.13	
2920	0	171.24	-45.13	
2921	0	171.24	-46.13	
2922	0	172.1	-33.63	
2923	0	172.1	-34.63	
2924	0	172.1	-40.63	
2925	0	172.1	-41.63	
2926	0	172.1	-43.63	
2927	0	172.1	-44.63	
2928	0	172.97	-34.13	
2929	0	172.97	-35.13	
2930	0	172.97	-36.13	
2931	0	172.97	-39.13	
2932	0	172.97	-40.13	
2933	0	172.97	-41.13	
2934	0	172.97	-43.13	
2935	0	172.97	-44.13	
2936	0	173.83	-34.63	
2937	0	173.83	-35.63	
2938	0	173.83	-36.63	
2939	0	173.83	-37.63	
2940	0	173.83	-38.63	
2941	0	173.83	-39.63	
2942	0	173.83	-40.63	
2943	0	173.83	-41.63	
2944	0	173.83	-42.63	
2945	0	173.83	-43.63	
2946	0	174.7	-35.13	
2947	0	174.7	-36.13	
2948	0	174.7	-37.13	
2949	0	174.7	-38.13	
2950	0	174.7	-39.13	
2951	0	174.7	-40.13	
2952	0	174.7	-41.13	
2953	0	174.7	-42.13	
2954	0	175.57	-35.63	
2955	0	175.57	-36.63	
2956	0	175.57	-37.63	
2957	0	175.57	-40.63	
2958	0	175.57	-41.63	
2959	0	176.43	-36.13	
2960	0	176.43	-37.13	
2961	0	176.43	-38.13	
2962	0	176.43	-39.13	
2963	0	176.43	-40.13	
2964	0	176.43	-41.13	
2965	0	177.3	-37.63	
2966	0	177.3	-38.63	
2967	0	177.3	-39.63	
2968	0	177.3	-40.63	
2969	0	178.16	-37.13	
2970	0	178.16	-38.13	
2971	0	178.16	-39.13	
2972	0	178.16	-49.13	
2973	0	178.16	-50.13	
2974	0	179.03	-37.63	
2975	0	179.03	-47.63	
2976	0	179.03	-49.63	
2977	0	105.42	-10.13	
2978	0	105.42	-11.13	
2979	0	106.28	-10.63	
2980	0	96.76	-11.13	
2981	0	96.76	-12.13	
2982	0	167.77	-29.13	
2983	0	112.35	-25.13	
2984	0	112.35	-26.13	
2985	0	113.21	-21.63	
2986	0	113.21	-22.63	
2987	0	113.21	-23.63	
2988	0	113.21	-24.63	
2989	0	113.21	-25.63	
2990	0	113.21	-26.63	
2991	0	113.21	-27.63	
2992	0	113.21	-28.63	
2993	0	114.08	-21.13	
2994	0	114.08	-22.13	
2995	0	114.08	-23.13	
2996	0	114.08	-27.13	
2997	0	114.08	-28.13	
2998	0	114.08	-29.13	
2999	0	114.08	-30.13	
3000	0	114.94	-19.63	
3001	0	114.94	-20.63	
3002	0	114.94	-21.63	
3003	0	114.94	-28.63	
3004	0	114.94	-29.63	
3005	0	114.94	-30.63	
3006	0	114.94	-31.63	
3007	0	114.94	-32.63	
3008	0	114.94	-33.63	
3009	0	114.94	-34.63	
3010	0	115.81	-20.13	
3011	0	115.81	-31.13	
3012	0	115.81	-32.13	
3013	0	115.81	-33.13	
3014	0	115.81	-34.13	
3015	0	115.81	-35.13	
3016	0	116.68	-20.63	
3017	0	116.68	-34.63	
3018	0	116.68	-35.63	
3019	0	117.54	-20.13	
3020	0	117.54	-21.13	
3021	0	117.54	-35.13	
3022	0	118.41	-17.63	
3023	0	118.41	-19.63	
3024	0	118.41	-20.63	
3025	0	118.41	-34.63	
3026	0	118.41	-35.63	
3027	0	119.27	-17.13	
3028	0	119.27	-18.13	
3029	0	119.27	-19.13	
3030	0	119.27	-20.13	
3031	0	119.27	-34.13	
3032	0	119.27	-35.13	
3033	0	120.14	-19.63	
3034	0	120.14	-33.63	
3035	0	120.14	-34.63	
3036	0	121.01	-14.13	
3037	0	121.01	-19.13	
3038	0	121.01	-20.13	
3039	0	121.01	-34.13	
3040	0	121.87	-13.63	
3041	0	121.87	-14.63	
3042	0	121.87	-16.63	
3043	0	121.87	-17.63	
3044	0	121.87	-18.63	
3045	0	121.87	-33.63	
3046	0	121.87	-34.63	
3047	0	122.74	-12.13	
3048	0	122.74	-14.13	
3049	0	122.74	-15.13	
3050	0	122.74	-16.13	
3051	0	122.74	-17.13	
3052	0	122.74	-34.13	
3053	0	123.6	-12.63	
3054	0	123.6	-13.63	
3055	0	123.6	-14.63	
3056	0	123.6	-15.63	
3057	0	123.6	-33.63	
3058	0	123.6	-34.63	
3059	0	124.47	-14.13	
3060	0	124.47	-15.13	
3061	0	124.47	-33.13	
3062	0	124.47	-34.13	
3063	0	125.34	-13.63	
3064	0	125.34	-14.63	
3065	0	125.34	-32.63	
3066	0	126.2	-13.13	
3067	0	126.2	-14.13	
3068	0	126.2	-32.13	
3069	0	127.07	-13.63	
3070	0	127.07	-32.63	
3071	0	127.93	-14.13	
3072	0	127.93	-15.13	
3073	0	127.93	-32.13	
3074	0	128.8	-13.63	
3075	0	128.8	-14.63	
3076	0	128.8	-31.63	
3077	0	129.67	-11.13	
3078	0	129.67	-12.13	
3079	0	129.67	-13.13	
3080	0	129.67	-14.13	
3081	0	129.67	-31.13	
3082	0	129.67	-32.13	
3083	0	130.53	-10.63	
3084	0	130.53	-11.63	
3085	0	130.53	-12.63	
3086	0	130.53	-31.63	
3087	0	131.4	-11.13	
3088	0	131.4	-31.13	
3089	0	131.4	-32.13	
3090	0	132.26	-10.63	
3091	0	132.26	-31.63	
3092	0	132.26	-32.63	
3093	0	133.13	-11.13	
3094	0	133.13	-12.13	
3095	0	133.13	-32.13	
3096	0	133.13	-33.13	
3097	0	134	-11.63	
3098	0	134	-12.63	
3099	0	134	-32.63	
3100	0	134	-33.63	
3101	0	134	-34.63	
3102	0	134.86	-11.13	
3103	0	134.86	-12.13	
3104	0	134.86	-33.13	
3105	0	134.86	-34.13	
3106	0	134.86	-35.13	
3107	0	135.73	-11.63	
3108	0	135.73	-14.63	
3109	0	135.73	-15.63	
3110	0	135.73	-34.63	
3111	0	135.73	-35.63	
3112	0	136.59	-11.13	
3113	0	136.59	-12.13	
3114	0	136.59	-13.13	
3115	0	136.59	-14.13	
3116	0	136.59	-15.13	
3117	0	136.59	-16.13	
3118	0	136.59	-36.13	
3119	0	137.46	-11.63	
3120	0	137.46	-12.63	
3121	0	137.46	-13.63	
3122	0	137.46	-14.63	
3123	0	137.46	-15.63	
3124	0	137.46	-16.63	
3125	0	137.46	-35.63	
3126	0	137.46	-36.63	
3127	0	138.33	-16.13	
3128	0	138.33	-17.13	
3129	0	138.33	-35.13	
3130	0	138.33	-36.13	
3131	0	139.19	-15.63	
3132	0	139.19	-16.63	
3133	0	139.19	-17.63	
3134	0	139.19	-35.63	
3135	0	139.19	-36.63	
3136	0	139.19	-37.63	
3137	0	140.06	-16.13	
3138	0	140.06	-17.13	
3139	0	140.06	-18.13	
3140	0	140.06	-36.13	
3141	0	140.06	-37.13	
3142	0	140.06	-38.13	
3143	0	140.92	-12.63	
3144	0	140.92	-13.63	
3145	0	140.92	-14.63	
3146	0	140.92	-15.63	
3147	0	140.92	-16.63	
3148	0	140.92	-17.63	
3149	0	140.92	-37.63	
3150	0	140.92	-38.63	
3151	0	141.79	-10.13	
3152	0	141.79	-11.13	
3153	0	141.79	-12.13	
3154	0	141.79	-13.13	
3155	0	141.79	-14.13	
3156	0	141.79	-15.13	
3157	0	141.79	-16.13	
3158	0	141.79	-38.13	
3159	0	141.79	-39.13	
3160	0	142.66	-10.63	
3161	0	142.66	-11.63	
3162	0	142.66	-38.63	
3163	0	143.52	-10.13	
3164	0	143.52	-11.13	
3165	0	143.52	-12.13	
3166	0	143.52	-13.13	
3167	0	143.52	-39.13	
3168	0	143.52	-40.13	
3169	0	144.39	-9.63	
3170	0	144.39	-10.63	
3171	0	144.39	-11.63	
3172	0	144.39	-12.63	
3173	0	144.39	-13.63	
3174	0	144.39	-14.63	
3175	0	144.39	-38.63	
3176	0	144.39	-39.63	
3177	0	144.39	-40.63	
3178	0	144.39	-41.63	
3179	0	144.39	-42.63	
3180	0	145.25	-14.13	
3181	0	145.25	-15.13	
3182	0	145.25	-16.13	
3183	0	145.25	-17.13	
3184	0	145.25	-38.13	
3185	0	145.25	-39.13	
3186	0	145.25	-40.13	
3187	0	145.25	-41.13	
3188	0	145.25	-42.13	
3189	0	145.25	-43.13	
3190	0	146.12	-14.63	
3191	0	146.12	-15.63	
3192	0	146.12	-16.63	
3193	0	146.12	-17.63	
3194	0	146.12	-18.63	
3195	0	146.12	-38.63	
3196	0	146.12	-39.63	
3197	0	146.12	-40.63	
3198	0	146.12	-41.63	
3199	0	146.12	-43.63	
3200	0	146.99	-17.13	
3201	0	146.99	-18.13	
3202	0	146.99	-19.13	
3203	0	146.99	-38.13	
3204	0	146.99	-39.13	
3205	0	146.99	-40.13	
3206	0	146.99	-41.13	
3207	0	146.99	-43.13	
3208	0	146.99	-44.13	
3209	0	147.85	-16.63	
3210	0	147.85	-17.63	
3211	0	147.85	-18.63	
3212	0	147.85	-19.63	
3213	0	147.85	-37.63	
3214	0	147.85	-38.63	
3215	0	147.85	-39.63	
3216	0	147.85	-40.63	
3217	0	147.85	-41.63	
3218	0	147.85	-42.63	
3219	0	147.85	-43.63	
3220	0	148.72	-17.13	
3221	0	148.72	-18.13	
3222	0	148.72	-19.13	
3223	0	148.72	-20.13	
3224	0	148.72	-38.13	
3225	0	148.72	-40.13	
3226	0	148.72	-41.13	
3227	0	148.72	-42.13	
3228	0	148.72	-43.13	
3229	0	149.58	-15.63	
3230	0	149.58	-16.63	
3231	0	149.58	-17.63	
3232	0	149.58	-19.63	
3233	0	149.58	-20.63	
3234	0	149.58	-35.63	
3235	0	149.58	-36.63	
3236	0	149.58	-37.63	
3237	0	150.45	-16.13	
3238	0	150.45	-17.13	
3239	0	150.45	-18.13	
3240	0	150.45	-21.13	
3241	0	150.45	-22.13	
3242	0	150.45	-23.13	
3243	0	150.45	-34.13	
3244	0	150.45	-35.13	
3245	0	150.45	-36.13	
3246	0	150.45	-37.13	
3247	0	151.32	-16.63	
3248	0	151.32	-17.63	
3249	0	151.32	-20.63	
3250	0	151.32	-21.63	
3251	0	151.32	-22.63	
3252	0	151.32	-23.63	
3253	0	151.32	-32.63	
3254	0	151.32	-33.63	
3255	0	151.32	-34.63	
3256	0	152.18	-17.13	
3257	0	152.18	-19.13	
3258	0	152.18	-21.13	
3259	0	152.18	-22.13	
3260	0	152.18	-23.13	
3261	0	152.18	-24.13	
3262	0	152.18	-25.13	
3263	0	152.18	-32.13	
3264	0	152.18	-33.13	
3265	0	153.05	-20.63	
3266	0	153.05	-21.63	
3267	0	153.05	-22.63	
3268	0	153.05	-23.63	
3269	0	153.05	-24.63	
3270	0	153.05	-25.63	
3271	0	153.05	-26.63	
3272	0	153.05	-27.63	
3273	0	153.05	-28.63	
3274	0	153.05	-29.63	
3275	0	153.05	-30.63	
3276	0	153.05	-31.63	
3277	0	153.05	-32.63	
3278	0	153.91	-21.13	
3279	0	153.91	-22.13	
3280	0	153.91	-25.13	
3281	0	153.91	-27.13	
3282	0	153.91	-28.13	
3283	0	153.91	-29.13	
3284	0	153.91	-30.13	
3285	0	154.78	-20.63	
3286	0	154.78	-21.63	
3287	0	154.78	-22.63	
3288	0	155.65	-17.13	
3289	0	155.65	-18.13	
3290	0	155.65	-21.13	
3291	0	155.65	-22.13	
3292	0	155.65	-23.13	
3293	0	158.24	-29.63	
3294	0	158.24	-31.63	
3295	0	159.11	-29.13	
3296	0	159.11	-30.13	
3297	0	159.11	-31.13	
3298	0	159.11	-32.13	
3299	0	158.24	-54.63	
3300	0	158.24	-55.63	
3301	0	159.11	-54.13	
3302	0	159.11	-55.13	
3303	0	114.08	4.87	
3304	0	114.94	5.37	
3305	0	114.94	4.37	
3306	0	98.49	5.87	
3307	0	99.36	5.37	
3308	0	99.36	4.37	
3309	0	100.22	3.87	
3310	0	100.22	2.87	
3311	0	101.09	3.37	
3312	0	101.09	2.37	
3313	0	101.95	2.87	
3314	0	101.95	1.87	
3315	0	102.82	6.37	
3316	0	102.82	5.37	
3317	0	102.82	1.37	
3318	0	103.69	5.87	
3319	0	103.69	4.87	
3320	0	103.69	3.87	
3321	0	103.69	2.87	
3322	0	104.55	3.37	
3323	0	104.55	2.37	
3324	0	109.75	2.37	
3325	0	110.61	2.87	
3326	0	110.61	1.87	
3327	0	111.48	3.37	
3328	0	111.48	2.37	
3329	0	112.35	3.87	
3330	0	112.35	2.87	
3331	0	113.21	4.37	
3332	0	113.21	3.37	
3333	0	114.94	6.37	
3334	0	115.81	6.87	
3335	0	115.81	5.87	
3336	0	117.54	5.87	
3337	0	117.54	3.87	
3338	0	118.41	4.37	
3339	0	103.69	8.87	
3340	0	104.55	8.37	
3341	0	105.42	19.87	
3342	0	105.42	18.87	
3343	0	105.42	8.87	
3344	0	105.42	7.87	
3345	0	106.28	21.37	
3346	0	106.28	20.37	
3347	0	106.28	19.37	
3348	0	106.28	18.37	
3349	0	106.28	17.37	
3350	0	106.28	8.37	
3351	0	107.15	20.87	
3352	0	107.15	19.87	
3353	0	107.15	17.87	
3354	0	107.15	16.87	
3355	0	107.15	8.87	
3356	0	108.02	21.37	
3357	0	108.02	20.37	
3358	0	108.02	17.37	
3359	0	108.02	16.37	
3360	0	108.02	9.37	
3361	0	108.88	15.87	
3362	0	108.88	14.87	
3363	0	108.88	13.87	
3364	0	108.88	12.87	
3365	0	108.88	11.87	
3366	0	108.88	10.87	
3367	0	108.88	9.87	
3368	0	109.75	15.37	
3369	0	109.75	14.37	
3370	0	109.75	13.37	
3371	0	109.75	12.37	
3372	0	109.75	11.37	
3373	0	109.75	10.37	
3374	0	108.02	19.37	
3375	0	108.02	18.37	
3376	0	108.88	21.87	
3377	0	108.88	20.87	
3378	0	108.88	19.87	
3379	0	108.88	18.87	
3380	0	108.88	17.87	
3381	0	109.75	21.37	
3382	0	109.75	20.37	
3383	0	109.75	19.37	
3384	0	109.75	18.37	
3385	0	110.61	19.87	
3386	0	110.61	18.87	
3387	0	110.61	17.87	
3388	0	111.48	20.37	
3389	0	111.48	19.37	
3390	0	112.35	21.87	
3391	0	112.35	20.87	
3392	0	113.21	21.37	
3393	0	114.08	21.87	
3394	0	114.94	22.37	
3395	0	115.81	22.87	
3396	0	115.81	21.87	
3397	0	116.68	23.37	
3398	0	116.68	22.37	
3399	0	117.54	39.87	
3400	0	117.54	38.87	
3401	0	117.54	37.87	
3402	0	117.54	23.87	
3403	0	117.54	22.87	
3404	0	118.41	39.37	
3405	0	118.41	38.37	
3406	0	118.41	37.37	
3407	0	118.41	24.37	
3408	0	118.41	23.37	
3409	0	119.27	39.87	
3410	0	119.27	38.87	
3411	0	119.27	37.87	
3412	0	119.27	36.87	
3413	0	119.27	24.87	
3414	0	120.14	40.37	
3415	0	120.14	39.37	
3416	0	120.14	38.37	
3417	0	120.14	37.37	
3418	0	120.14	35.37	
3419	0	120.14	34.37	
3420	0	120.14	26.37	
3421	0	120.14	25.37	
3422	0	121.01	40.87	
3423	0	121.01	39.87	
3424	0	121.01	38.87	
3425	0	121.01	37.87	
3426	0	121.01	35.87	
3427	0	121.01	33.87	
3428	0	121.01	32.87	
3429	0	121.01	27.87	
3430	0	121.01	26.87	
3431	0	121.87	41.37	
3432	0	121.87	40.37	
3433	0	121.87	39.37	
3434	0	121.87	38.37	
3435	0	121.87	37.37	
3436	0	121.87	36.37	
3437	0	121.87	33.37	
3438	0	121.87	32.37	
3439	0	121.87	31.37	
3440	0	121.87	29.37	
3441	0	121.87	28.37	
3442	0	121.87	27.37	
3443	0	122.74	40.87	
3444	0	122.74	39.87	
3445	0	122.74	38.87	
3446	0	122.74	37.87	
3447	0	122.74	36.87	
3448	0	122.74	31.87	
3449	0	122.74	30.87	
3450	0	122.74	29.87	
3451	0	122.74	28.87	
3452	0	123.6	40.37	
3453	0	123.6	30.37	
3454	637	278.36	-4.13	
3455	673	278.36	-5.13	
3456	1184	278.36	-6.13	
3457	0	279.22	-3.63	
3458	0	279.22	-5.63	
3459	283	279.22	-6.63	
3460	803	279.22	-7.63	
3461	0	280.09	-7.13	
3462	127	280.09	-8.13	
3463	0	280.95	-7.63	
3464	25	280.95	-8.63	
3465	212	280.95	-9.63	
3466	0	281.82	-9.13	
3467	34	281.82	-10.13	
3468	93	281.82	-11.13	
3469	287	281.82	-12.13	
3470	0	282.69	-11.63	
3471	63	282.69	-12.63	
3472	24	283.55	-13.13	
3473	95	283.55	-14.13	
3474	44	283.55	-15.13	
3475	0	284.42	-14.63	
3476	19	284.42	-15.63	
3477	0	285.28	-15.13	
3478	6	285.28	-16.13	
3479	0	286.15	-15.63	
3480	3	286.15	-16.63	
3481	0	287.02	-16.13	
3482	4	287.02	-17.13	
3483	0	287.88	-16.63	
3484	0	287.88	-17.63	
3485	0	288.75	-17.13	
3486	1	288.75	-18.13	
3487	0	122.74	24.87	
3488	0	122.74	23.87	
3489	0	123.6	24.37	
3490	0	123.6	23.37	
3491	0	124.47	24.87	
3492	0	124.47	23.87	
3493	0	125.34	25.37	
3494	0	125.34	24.37	
3495	0	126.2	26.87	
3496	0	126.2	25.87	
3497	0	127.07	26.37	
3498	0	127.93	32.87	
3499	0	127.93	31.87	
3500	0	127.93	27.87	
3501	0	127.93	26.87	
3502	0	127.93	25.87	
3503	0	128.8	33.37	
3504	0	128.8	32.37	
3505	0	128.8	31.37	
3506	0	128.8	30.37	
3507	0	128.8	29.37	
3508	0	128.8	28.37	
3509	0	128.8	27.37	
3510	1	128.8	26.37	
3511	0	129.67	33.87	
3512	0	129.67	31.87	
3513	0	129.67	30.87	
3514	0	129.67	29.87	
3515	0	129.67	28.87	
3516	0	129.67	27.87	
3517	0	130.53	35.37	
3518	0	130.53	34.37	
3519	0	130.53	31.37	
3520	0	130.53	30.37	
3521	3	130.53	28.37	
3522	0	130.53	24.37	
3523	0	131.4	34.87	
3524	0	131.4	32.87	
3525	0	131.4	31.87	
3526	5	131.4	30.87	
3527	11	131.4	29.87	
3528	3	131.4	25.87	
3529	1	131.4	24.87	
3530	0	131.4	23.87	
3531	0	132.26	36.37	
3532	0	132.26	35.37	
3533	3	132.26	32.37	
3534	0	133.13	36.87	
3535	0	133.13	35.87	
3536	2	133.13	32.87	
3537	0	134	36.37	
3538	0	134	35.37	
3539	0	134	33.37	
3540	0	134.86	35.87	
3541	0	134.86	33.87	
3542	0	135.73	36.37	
3543	0	135.73	33.37	
3544	14	135.73	20.37	
3545	0	136.59	37.87	
3546	0	136.59	36.87	
3547	0	136.59	33.87	
3548	0	137.46	38.37	
3549	0	137.46	34.37	
3550	0	138.33	38.87	
3551	0	138.33	37.87	
3552	0	138.33	34.87	
3553	0	138.33	33.87	
3554	0	139.19	42.37	
3555	0	139.19	41.37	
3556	0	139.19	40.37	
3557	0	139.19	39.37	
3558	0	139.19	38.37	
3559	391	139.19	34.37	
3560	150	139.19	33.37	
3561	288	139.19	32.37	
3562	140	139.19	31.37	
3563	0	140.06	43.87	
3564	0	140.06	42.87	
3565	0	140.06	41.87	
3566	0	140.06	40.87	
3567	0	140.06	35.87	
3568	6345	140.06	34.87	
3569	24415	140.06	33.87	
3570	18601	140.06	32.87	
3571	16088	140.06	31.87	
3572	3072	140.06	30.87	
3573	237	140.06	29.87	
3574	0	140.92	45.37	
3575	0	140.92	44.37	
3576	0	140.92	43.37	
3577	0	140.92	42.37	
3578	0	140.92	41.37	
3579	0	140.92	40.37	
3580	0	140.92	38.37	
3581	1415	140.92	37.37	
3582	5731	140.92	36.37	
3583	44336	140.92	35.37	
3584	3331	140.92	30.37	
3585	85	140.92	27.37	
3586	18	140.92	25.37	
3587	12	140.92	24.37	
3588	0	141.79	44.87	
3589	0	141.79	43.87	
3590	0	141.79	42.87	
3591	2	141.79	41.87	
3592	1178	141.79	40.87	
3593	303	141.79	39.87	
3594	1959	141.79	38.87	
3595	7692	141.79	37.87	
3596	467	141.79	27.87	
3597	1	141.79	26.87	
3598	84	141.79	25.87	
3599	211	141.79	24.87	
3600	143	141.79	23.87	
3601	0	142.66	45.37	
3602	0	142.66	44.37	
3603	1	142.66	42.37	
3604	3280	142.66	41.37	
3605	9048	142.66	39.37	
3606	724	142.66	27.37	
3607	268	142.66	26.37	
3608	1	143.52	44.87	
3609	0	143.52	43.87	
3610	0	143.52	42.87	
3611	4102	143.52	41.87	
3612	1	144.39	44.37	
3613	0	144.39	43.37	
3614	3101	144.39	42.37	
3615	490	145.25	44.87	
3616	32	145.25	43.87	
3617	2674	145.25	42.87	
3618	2172	146.12	43.37	
3619	1496	153.91	24.87	
3620	1138	153.91	23.87	
3621	0	95.02	5.87	
3622	0	95.02	4.87	
3623	0	95.02	3.87	
3624	0	95.02	2.87	
3625	0	95.89	6.37	
3626	0	95.89	5.37	
3627	0	95.89	2.37	
3628	0	96.76	5.87	
3629	0	96.76	4.87	
3630	0	96.76	1.87	
3631	0	96.76	0.87	
3632	0	97.62	5.37	
3633	0	97.62	4.37	
3634	0	97.62	0.37	
3635	0	97.62	-0.63	
3636	0	98.49	4.87	
3637	0	98.49	3.87	
3638	0	98.49	-1.13	
3639	0	98.49	-2.13	
3640	0	99.36	3.37	
3641	0	99.36	-1.63	
3642	0	99.36	-2.63	
3643	0	100.22	-3.13	
3644	0	100.22	-4.13	
3645	0	101.09	-3.63	
3646	0	101.09	-4.63	
3647	0	101.95	-5.13	
3648	0	101.95	-6.13	
3649	0	102.82	-5.63	
3650	0	103.69	-5.13	
3651	0	103.69	-6.13	
3652	0	104.55	-5.63	
3653	0	104.55	-6.63	
3654	0	105.42	3.87	
3655	0	105.42	2.87	
3656	0	105.42	1.87	
3657	0	105.42	-7.13	
3658	0	106.28	4.37	
3659	0	106.28	3.37	
3660	0	106.28	-7.63	
3661	0	107.15	4.87	
3662	0	107.15	3.87	
3663	0	107.15	-7.13	
3664	0	107.15	-8.13	
3665	0	108.02	5.37	
3666	0	108.02	4.37	
3667	0	108.02	-7.63	
3668	0	108.88	3.87	
3669	0	108.88	2.87	
3670	0	108.88	-8.13	
3671	0	109.75	-7.63	
3672	0	109.75	-8.63	
3673	0	110.61	-8.13	
3674	0	111.48	-8.63	
3675	0	112.35	-8.13	
3676	0	112.35	-9.13	
3677	0	113.21	-8.63	
3678	0	114.08	-9.13	
3679	0	114.94	-8.63	
3680	0	115.81	-9.13	
3681	0	116.68	-8.63	
3682	0	116.68	-9.63	
3683	0	117.54	-9.13	
3684	0	118.41	3.37	
3685	0	118.41	2.37	
3686	0	118.41	-8.63	
3687	0	118.41	-9.63	
3688	0	119.27	1.87	
3689	0	119.27	-10.13	
3690	0	120.14	1.37	
3691	0	120.14	-10.63	
3692	0	121.01	1.87	
3693	0	121.01	0.87	
3694	0	121.01	-10.13	
3695	0	121.01	-11.13	
3696	0	121.87	1.37	
3697	0	121.87	-10.63	
3698	0	122.74	0.87	
3699	0	122.74	-11.13	
3700	0	123.6	1.37	
3701	0	123.6	-10.63	
3702	0	124.47	3.87	
3703	0	124.47	2.87	
3704	0	124.47	1.87	
3705	0	124.47	0.87	
3706	0	124.47	-8.13	
3707	0	124.47	-10.13	
3708	0	125.34	4.37	
3709	0	125.34	3.37	
3710	0	125.34	2.37	
3711	0	125.34	-7.63	
3712	0	125.34	-8.63	
3713	0	125.34	-9.63	
3714	0	126.2	4.87	
3715	0	126.2	-3.13	
3716	0	126.2	-8.13	
3717	0	127.07	5.37	
3718	0	127.07	4.37	
3719	0	127.07	3.37	
3720	0	127.07	-7.63	
3721	0	127.07	-8.63	
3722	0	127.93	3.87	
3723	0	127.93	2.87	
3724	0	127.93	-8.13	
3725	0	128.8	2.37	
3726	0	128.8	1.37	
3727	0	128.8	0.37	
3728	0	128.8	-8.63	
3729	0	129.67	0.87	
3730	0	129.67	-8.13	
3731	0	130.53	1.37	
3732	0	130.53	0.37	
3733	0	130.53	-8.63	
3734	0	131.4	0.87	
3735	0	131.4	-0.13	
3736	0	131.4	-7.13	
3737	0	131.4	-8.13	
3738	0	132.26	0.37	
3739	0	132.26	-0.63	
3740	0	132.26	-3.63	
3741	0	132.26	-6.63	
3742	0	132.26	-7.63	
3743	0	133.13	0.87	
3744	0	133.13	-0.13	
3745	0	133.13	-3.13	
3746	0	133.13	-6.13	
3747	0	133.13	-7.13	
3748	0	134	1.37	
3749	0	134	0.37	
3750	0	134	-6.63	
3751	0	134	-7.63	
3752	0	134.86	0.87	
3753	0	134.86	-0.13	
3754	0	134.86	-5.13	
3755	0	134.86	-6.13	
3756	0	134.86	-7.13	
3757	0	135.73	-0.63	
3758	0	135.73	-4.63	
3759	0	135.73	-5.63	
3760	0	136.59	-1.13	
3761	0	136.59	-5.13	
3762	0	137.46	-0.63	
3763	0	137.46	-1.63	
3764	0	137.46	-5.63	
3765	0	137.46	-6.63	
3766	0	137.46	-7.63	
3767	0	137.46	-8.63	
3768	0	138.33	-1.13	
3769	0	138.33	-6.13	
3770	0	138.33	-7.13	
3771	0	138.33	-8.13	
3772	0	138.33	-9.13	
3773	0	139.19	-1.63	
3774	0	139.19	-8.63	
3775	0	140.06	-2.13	
3776	0	140.06	-8.13	
3777	0	140.06	-9.13	
3778	0	140.92	-8.63	
3779	0	126.2	-9.13	
3780	0	294.81	32.37	
3781	0	295.68	32.87	
3782	0	295.68	31.87	
