# Supplementary material for: A predictive framework for identifying source populations of non-native marine macroalgae: Chondria tumulosa in the Pacific Ocean
Source: PeerJ. 2025 Jun 23;13:e19610. doi: 10.7717/peerj.19610 (PMC12199741; doi:10.7717/peerj.19610)
Supplement: Supplemental Information 10 — Displayed p-values represent those from Japan/The central Pacific/the Eastern Tropical Pacific. Significance is indicated by an asterisk. [file peerj-13-19610-s010.rtf]

	El Niño 	La Niña 	Neutral	
La Niña 	1.0/ 1.0/ 0.44	–	–	
Neutral	0.038*/ 1.0/ 0.31	0.003*/ 0.24/1.0	–	
