# Supplementary material for: A predictive framework for identifying source populations of non-native marine macroalgae: Chondria tumulosa in the Pacific Ocean
Source: PeerJ. 2025 Jun 23;13:e19610. doi: 10.7717/peerj.19610 (PMC12199741; doi:10.7717/peerj.19610)
Supplement: Supplemental Information 11 — Displayed p-values represent those from Japan/the central Pacific/the eastern Pacific. Seasons are December, January, and February (DJF); March, April, and May (MAM); June, July, and August (JJA), and September, October, and November (SON). [file peerj-13-19610-s011.rtf]

	DJF	JJA	MAM	
JJA	1.0/ 1.0/ 1.0	–	–	
MAM	0.99/ 1.0/ 1.0	0.45/ 1.0/ 1.0	–	
SON	1.0/ 1.0/ 0.41	1.0/ 1.0/ 1.0	1.0/ 1.0/ 1.0	
