# Supplementary material for: A predictive framework for identifying source populations of non-native marine macroalgae: Chondria tumulosa in the Pacific Ocean
Source: PeerJ. 2025 Jun 23;13:e19610. doi: 10.7717/peerj.19610 (PMC12199741; doi:10.7717/peerj.19610)
Supplement: Supplemental Information 12 — Significance is indicated by an asterisk. [file peerj-13-19610-s012.rtf]

	0.5 m2/s	5 m2/s	10 m2/s	20 m2/s	
5 m2/s	2.8e-10*	–	–	–	
10 m2/s	1.7e-15*	0.264	–	–	
20 m2/s	3.1e-13*	1.0	1.0	–	
50 m2/s	4.7e-7*	1.0	0.081	0.285	
